# Sarcoidosis: Updates on therapeutic drug trials and novel treatment approaches

**DOI:** 10.3389/fmed.2022.991783

**Published:** 2022-10-12

**Authors:** Ogugua Ndili Obi, Lesley Ann Saketkoo, Anne-Marie Russell, Robert P. Baughman

**Affiliations:** ^1^Division of Pulmonary Critical Care and Sleep Medicine, Brody School of Medicine, East Carolina University, Greenville, NC, United States; ^2^New Orleans Scleroderma and Sarcoidosis Patient Care and Research Center, New Orleans, LA, United States; ^3^University Medical Center—Comprehensive Pulmonary Hypertension Center and Interstitial Lung Disease Clinic Programs, New Orleans, LA, United States; ^4^Section of Pulmonary Medicine, Louisiana State University School of Medicine, New Orleans, LA, United States; ^5^Department of Undergraduate Honors, Tulane University School of Medicine, New Orleans, LA, United States; ^6^Exeter Respiratory Institute University of Exeter, Exeter, United Kingdom; ^7^Royal Devon and Exeter NHS Foundation Trust, Devon, United Kingdom; ^8^Faculty of Medicine, Imperial College and Imperial College Healthcare NHS Trust, London, United Kingdom; ^9^Department of Medicine, University of Cincinnati, Cincinnati, OH, United States

**Keywords:** pulmonary sarcoidosis, progressive pulmonary fibrosis, fibrotic pulmonary sarcoidosis, patient centered and patient partners in research, interstitial lung disease, therapeutic pathways, clinical trials and clinical trial design, novel therapies

## Abstract

Sarcoidosis is a systemic granulomatous inflammatory disease of unknown etiology. It affects the lungs in over 90% of patients yet extra-pulmonary and multi-organ involvement is common. Spontaneous remission of disease occurs commonly, nonetheless, over 50% of patients will require treatment and up to 30% of patients will develop a chronic progressive non-remitting disease with marked pulmonary fibrosis leading to significant morbidity and death. Guidelines outlining an immunosuppressive treatment approach to sarcoidosis were recently published, however, the strength of evidence behind many of the guideline recommended drugs is weak. None of the drugs currently used for the treatment of sarcoidosis have been rigorously studied and prescription of these drugs is often based on off-label” indications informed by experience with other diseases. Indeed, only two medications [prednisone and repository corticotropin (RCI) injection] currently used in the treatment of sarcoidosis are approved by the United States Food and Drug Administration. This situation results in significant reimbursement challenges especially for the more advanced (and often more effective) drugs that are favored for severe and refractory forms of disease causing an over-reliance on corticosteroids known to be associated with significant dose and duration dependent toxicities. This past decade has seen a renewed interest in developing new drugs and exploring novel therapeutic pathways for the treatment of sarcoidosis. Several of these trials are active randomized controlled trials (RCTs) designed to recruit relatively large numbers of patients with a goal to determine the safety, efficacy, and tolerability of these new molecules and therapeutic approaches. While it is an exciting time, it is also necessary to exercise caution. Resources including research dollars and most importantly, patient populations available for trials are limited and thus necessitate that several of the challenges facing drug trials and drug development in sarcoidosis are addressed. This will ensure that currently available resources are judiciously utilized. Our paper reviews the ongoing and anticipated drug trials in sarcoidosis and addresses the challenges facing these and future trials. We also review several recently completed trials and draw lessons that should be applied in future.

## Introduction

Sarcoidosis is a systemic inflammatory disease of unknown etiology characterized by the presence of non-caseating granulomas in affected organs (1). The lungs are affected in over 90% of patients yet extra-pulmonary and multi-organ involvement occurs commonly (2, 3). The clinical presentation, disease course, and severity of sarcoidosis is highly variable, impacting treatment, prognosis, and patient outcomes (1, 4, 5). A good proportion of patients will have spontaneous disease remission, up to 50% of patients will require treatment, and 10–30% of patients will develop a chronic unremitting disease with in some cases marked pulmonary fibrosis and varying degrees of respiratory failure (5–9). Approximately 5% of patients with sarcoidosis die from their disease with higher mortality reported in the population of patients with respiratory failure, fibrotic pulmonary disease, pulmonary hypertension, and cardiac sarcoidosis (CS) (10–16).

None of the drugs currently used for sarcoidosis treatment have been rigorously studied in large randomized controlled trials (RCTs) (5, 17). Most drugs used in sarcoidosis treatment are prescribed on “off-label” indications informed by experience with other diseases. Indeed, sarcoidosis treatment is based on results from trials whose design and methods suffer from inherent trial design flaws of rarer conditions including sample size, selection for active disease, and clinically meaningful endpoints that include validated patient-reported outcome measures (PROMs). Where large studies exist, they have been focused on pulmonary sarcoidosis to the neglect of other organ-threatening extra-pulmonary disease manifestations (17). This presents several challenges to drug acquisition especially for the more advanced drugs that are favored for severe/refractory forms of sarcoidosis (5, 18, 19) and are frequently denied by reimbursement agencies because sarcoidosis is not listed as an FDA approved indication for use. The reasons for this are many. First, sarcoidosis is considered a rare disease with relatively few people affected and even fewer (50–80% of those affected) potentially needing treatment (3, 8, 20). This impacts disease awareness, research funding and severely strains the pool of patients eligible for clinical trials especially in non-pulmonary disease manifestations. Secondly, there is no widely accepted biological model of disease thus limiting the scope and rate of pre-clinical drug development. Thirdly, sarcoidosis is a very heterogenous disease with a highly variable disease course and a lack/scarcity of available validated active disease measures. Therefore, great challenges in sarcoidosis trial design are to adequately define a target study group and the availability of standardized outcome measures that accurately measure disease responsiveness while maintaining a patient-centered focus (5, 21, 22).

The recently published European Respiratory Society (ERS) clinical practice guidelines help to address these concerns by outlining tentative treatment approaches for various organ manifestations of sarcoidosis (5). Very importantly, the guidelines reaffirmed two major reasons to initiate treatment in sarcoidosis patients: to lower the morbidity and mortality risk associated with sarcoidosis or to improve quality of life (QoL) largely related to symptom burden and decline in physical function due to disease (HRQoL) (5). Although a major step in the right direction, the ERS guidelines were developed as a general guidance in response to presumed historical clinical practice and all 12 treatment recommendations were associated with a level of evidence deemed very low to low quality (5). Furthermore, the guidelines do not address all the concerns surrounding medication prescription, and do not eliminate the barriers surrounding medication acquisition. The guidelines also do not address an increasingly common practice of “hit hard and early” whereby more and more sarcoidosis physicians are combining steroids and steroid-sparing medications up front in severe manifestations of disease (5).

Current advancements in personalized and precision medicine as well as the introduction of compounds developed specifically for sarcoidosis treatment, underscore the imperative of standardized elements of clinical trial design in either collective or organ-specific sarcoidosis. Medications used in sarcoidosis warrant rigorous, methodical studies targeted to the patients for whom their use is intended. Nearly 50% of patients requiring therapy for severe forms of sarcoidosis may experience a therapeutic failure (toxicity, intolerability, or inefficacy) (23), making it crucial that a pipeline of rigorously evaluated drugs can continue to be deployed.

This manuscript will review the current and anticipated drug trials in sarcoidosis with a focus on studies evaluating novel molecules and novel therapeutic pathways. Trials advocating for a “hit hard and early approach” (24) and re-evaluating the current paradigm of “prednisone first followed by stepwise addition of steroid sparing agents” (25) will also be discussed. We will highlight several challenges that affect future and ongoing sarcoidosis drug trials and offer potential solutions to the most pressing needs. It is hoped that this manuscript will appeal to a wide readership audience that includes clinicians caring for patients, researchers, regulators, pharmaceutical industries sponsoring drug trials, and patients for whom these drugs are intended.

## Brief review of the currently available medications and treatment considerations in sarcoidosis

### Current landscape of systemic treatment

The treatment of sarcoidosis is not clear cut and demands rigorous ongoing attention. As noted above, the ERS guidelines reaffirmed two major reasons to initiate treatment (5). Unfortunately, the morbidity and mortality risk, and HRQoL impact associated with disease vary from one organ manifestation to another, and perhaps from one patient to another. Consequently, though well intended, the specifics of these concepts are subject to interpretation. Furthermore, there remains an inconsistency in the risk parameters and PROMs used to quantify these concepts (26). For patients with pulmonary sarcoidosis, initiation of systemic treatment is reserved for patients who communicate symptomatic disease impacting HRQoL (4, 5); and/or whether the patient’s disease can lead to progressive lung function decline or significant morbidity or mortality (4, 5, 27). For patients with extra-pulmonary involvement, the decision to treat is similarly dependent on the presence of clinically significant disease activity in the affected organ (presumed to impair HRQoL and/or threaten organ function) and is left to the clinicians judgment (5). Presence of clinically significant cardiac, neurologic, ocular, or renal involvement is often associated with significant morbidity and mortality, and treatment is usually indicated (5).

Although the guidelines and a previously published consensus study advocate for early introduction of steroid-sparing agents, there is no definition of how “early” these can be added (5, 7). Therefore, the treatment of sarcoidosis is fraught with myriad complexities along with diversity of comfort with the use of steroid-sparing immunosuppression which acculturates prescribing habits and necessitates shared decision-making (SDM) (28).

Corticosteroids have historically been the prototypical treatment in sarcoidosis, yet they are associated with a reduced HRQoL and significantly high morbidity and organ-threatening toxicities that are dependent on dose, duration and in some cases, genetic make-up (29–37). Patients with sarcoidosis on prolonged or high-dose steroids are more likely to be obese or overweight and have several endocrinological and cardiovascular adverse events (30, 32, 33). As prolonged use of corticosteroids is associated with significant organ-based and systemic toxicities without commensurable benefit to improved lung function (29–35), a dynamic treatment trial of corticosteroids for 3–6 months with proactive dose reduction to the minimal effective dose [of which the goal is < 10 mg daily (38, 39)] was mentioned as an acceptable rationale to limit the continuation and overuse of corticosteroids (5). It is hoped that this will ensure ongoing clinical evaluation to discriminate for the need to introduce alternate steroid-sparing therapy. Lack of vigilance of glucocorticoid use is common and is reported by both patients and researchers to be associated with adverse outcomes especially in non-whites and those of lower socioeconomic status (28, 40–45). In some sarcoidosis centers glucocorticoids are used as a bridge with concomitant weaning until steroid-sparing agents reach efficacious doses (28).

### Approaches to systemic treatment

The ERS guidelines and a recently published Delphi consensus statement from a large group of worldwide sarcoidosis experts advocate an approach to therapy that balances the use of reduced doses of corticosteroids with the (early) stepwise addition of steroid sparing anti-inflammatory non-biologic and biologic agents (5, 7).

The proposed approach to corticosteroid use is to limit continuation to a 3–6 month period to allow for demonstration of therapeutic response (7). During that time period, attempts are made to taper to the minimal effective dose with a goal maintenance dose of < 10 mg/day of prednisone/prednisone equivalent (7). If the patient’s disease remains uncontrolled on minimal steroid doses or significant steroid side effects develop, therapy is then stepped up to steroid-sparing non-biologic immunosuppressive therapy (IST) with further attempts made to wean corticosteroids to a prednisone equivalent dose of < 10 mg/day (5, 7). The guidelines make for early/concomitant initiation of steroid-sparing non-biologic agents (so-called “second-line agents”) for patients with CS or other forms of severe or multi-organ disease where prolonged therapy is anticipated, or where there is a high risk of steroid-induced toxicity (5, 7). For patients with symptomatic pulmonary sarcoidosis believed to be at higher risk of future mortality or permanent disability from sarcoidosis who have been treated with glucocorticoids and have continued disease or unacceptable side effects from glucocorticoids, the guidelines recommend the addition of methotrexate (5). Methotrexate is considered the preferred “second-line agent” with the most data supporting its use in sarcoidosis (5, 7, 46–48). Other commonly used “second-line agents” include: Azathioprine, Leflunomide, and Mycophenolate Mofetil, however, the evidence behind these latter medication recommendations is very weak (5).

**FIGURE 1:**
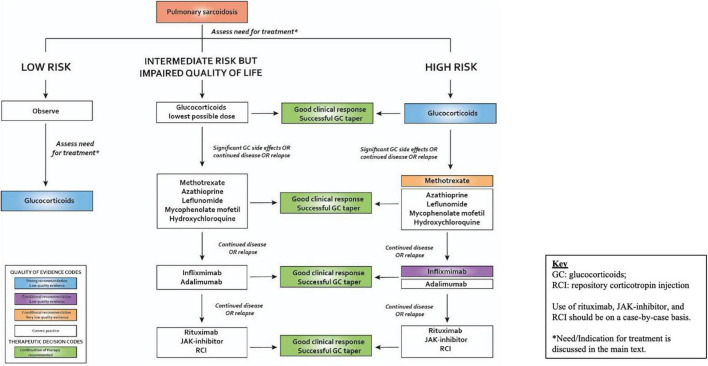
Approach to management of plumonary sarcoidosis. Reproduced with permission from the European Respiratory Society (ERS) clinical practice guidelines on the treatment of sarcoidosis (5). All rights reserved.

For patients with symptomatic pulmonary sarcoidosis believed to be at higher risk of future mortality or permanent disability from sarcoidosis who have been treated with glucocorticoids or other IST and have continued disease, the guidelines suggest the addition of infliximab to improve and/or preserve lung function and HRQoL (5). This was a conditional recommendation with overall low quality of evidence (5). Infliximab is a tumor necrosis factor inhibitor (TNFi) that has been shown to be effective in severe and refractory forms of pulmonary (49–62) and extra-pulmonary sarcoidosis (49, 63). TNFi (Infliximab and Adalimumab) have historically been regarded as “third-line agents” to be added in patients whose disease is uncontrolled on (or who develop significant toxicity to) “second-line therapy” (5, 7, 64). Infliximab has the most data supporting its use in sarcoidosis and is the preferred and more commonly prescribed “third-line agent” (5, 7, 64). Other advanced immunomodulating steroid-sparing agents suggested for use in patients with advanced and refractory disease include rituximab and Repository Corticotropin (RCI) (5). Practical suggestions and experience based recommendations on the use and management of TNFi in sarcoidosis have been published (64). The current widely accepted stepwise medications used in sarcoidosis are listed in Table 1 and the stepwise approach to treatment in pulmonary sarcoidosis is shown in Figure 1. Treatment algorithms specific to other organ manifestations have been published (5).

**TABLE 1:** Current widely accepted stepwise medications for the treatment of Sarcoidosis (5, 7, 48, 64).

Historic designation	Drug name	Usual dosage	Major toxicity	Drug monitoring	Comments
“First-Line”	Prednisone/ Prednisolone	20 mg/day initial dose, tapered to 5–10 mg QD to QoD	Weight gain, Diabetes Mellitus, Hypertension, Osteoporosis, Cataracts, Glaucoma, Sleep disturbance, Depression	Blood pressure and serum glucose monitoring, Bone density, Eye exams Body mass index	Causes cumulative toxicity that is dose and duration dependent.
“Second-Line” (Anti-metabolites)	Methotrexate	10–15 mg once a week PO Maybe given SQ if severe GI intolerance	GI Intolerance, Hepatotoxicity, Leukopenia, Fatigue, Pneumonitis.	CBC, LFT, renal function Folate supplementation is recommended.	Preferred anti-metabolite Teratogenic; avoid in pregnancy in both males and females of child-bearing age. Cleared by kidney, avoid in significant renal failure. Doses < 15 mg/week associated with inefficacy.
	Azathioprine	50–250 mg QD	Nausea, Leukopenia, Hepatotoxicity, Risk of Infections, Cutaneous and Lymphoproliferative Cancers.	CBC, LFT	Consider check TPMT level at initiation
	Leflunomide	10–20 mg QD	Nausea, Leukopenia, Hepatotoxicity, Peripheral Neuropathy, Pneumonitis	CBC, LFT, renal function	Due to long half-life, cholestyramine may be necessary to clear drug and its metabolites in toxicity. Teratogenic, avoid in pregnancy and breastfeeding. Cleared by kidney, avoid in significant renal failure
	Mycophenolate Mofetil	500–1,500 mg BID	Diarrhea, Leukopenia, risk of infections, Lymphoproliferative, and Cutaneous cancers	CBC, LFT Negative hepatitis B/C screening and negative IGRA are required prior to initiation	Less experience in sarcoidosis than other agents. Non-nephrotoxic
“Third-Line” Reserved for patients who have failed prior treatment with steroids and/or anti-metabolites	Infliximab or Biosimilars	3–5 mg/Kg IV at weeks 0, 2 and every 4–6weeks	Infections, allergic reactions. Contraindicated in demyelinating neurologic disease, active tuberculosis, deep fungal infections, prior malignancy, and severe CHF	Monitor for allergic reactions Screen for prior tuberculosis (negative IGRA testing) prior to initiation. Negative hepatitis B/C screening also advised.	Allergic reactions can be life threatening. Consider co-administration with Methotrexate to minimize formation of anti-drug antibodies.
	Adalimumab	40 mg SQ every 1–2 weeks	Infections, Allergic reactions Contraindicated in demyelinating neurologic disease, active tuberculosis, deep fungal infections, prior malignancy, and severe CHF	Monitor for allergic reactions Screen for prior tuberculosis (negative IGRA testing) prior to initiation. Negative hepatitis B/C screening also advised.	Less toxic than infliximab. Has been successfully used in patient’s intolerant to infliximab.
	Rituximab	500–1,000 mg IV every 1–6 months	Infections	Screen for viral hepatitis. Check IgG level with chronic therapy	High risk for viral reactivation. Can lead to IgG deficiency.
	Repository corticotropin Injection (RCI)	40–80 Units SQ twice a week	Diabetes Mellitus, Hypertension, Anxiety, Edema, Weight gain, Cataracts, Glaucoma, Sleep Disturbance.	Blood pressure and serum glucose monitoring, Bone density, Eye Exams Body Mass Index	Need to wean prednisone quickly to avoid cumulative toxicity.
Others	Hydroxychloroquine	200–400 mg QD	Loss of vision GI side effects,—abdominal pain, anorexia.	Regular eye exams depending on age and renal function	Beneficial for cutaneous disease. Minimal impact in cardiac and neurologic disease.

CBC, complete blood count; LFT, liver function test; IGRA, interferon gamma release assay for tuberculosis; PO, per oral; SQ, subcutaneously; IV, intravenously; QD, daily; QoD, every other day; TPMT, thiopurine S-methyltransferase (TPMT) genotype or enzyme activity; IgG, Immunoglobulin G; GI, Gastrointestinal (Intolerance, Nausea, vomiting, diarrhea); CHF, congestive heart failure.

### Non-pharmacological therapies and palliation of symptoms

The main focus of this paper is the implementation and investigation of pharmacologic systemic anti-inflammatory therapy, yet it must be noted that there are various non-systemic and non-pharmacologic therapies that have shown benefit in palliating symptoms. The intended use of systemic anti-inflammatory therapy is to halt or reverse active or partially active sarcoidosis along with remission of associated symptoms and physical impairment. Symptoms and physical impairment arising solely from irreversible tissue damage resulting from previously active disease is usually not amenable to anti-inflammatory treatment. Innumerable combinations of both pharmacological and non-pharmacological palliative measures exist to augment HRQoL by ameliorating organ-specific symptoms and physical impairment related to irreversible tissue damage (28). These include psychological, nutritional, strategic coping, mindfulness, physical/respiratory/occupational therapy, and possibly supportive pharmacology such as mucolytics, inhalers, anti-emetics etc. (28). Exercise and physical training are areas that are gaining momentum as systemic non-pharmacological treatment in inflammatory diseases and should be leveraged more frequently in sarcoidosis (65–69). Physical training/exercise is arguably both a systemic treatment that can modulate inflammation and a non-pharmacological therapy that cultivates physical capacity through amplification of neuromuscular and vascular networks and other bio-mechanical pathways that reduce symptom burden regardless of sarcoidosis disease activity status (67, 69–71).

### Treatment failure in sarcoidosis

Systemic medications offer hope for reversing the progression of moderate to severe disease activity and if successful provide the opportunity to re-gain global function as close to a person’s baseline as possible. As stated above, the systemic treatment armamentarium in sarcoidosis is limited, while the likelihood of treatment failure is reported to be fairly high (23). It is crucial to understand what constitutes treatment failure, the types of treatment failure, and whether the “failure” is salvageable. At the heart of treatment success and adherence, is SDM (72). SDM conveys knowledge that ties disease behavior to anticipated expectations of efficacy and considers side effects, potential toxicity and how toxicities are avoided (72, 73). SDM assesses and discusses a person’s treatment priorities, expectations and desires and has been shown to potentially influence response to therapy (74). Being a powerful component of clinical management, national cost-free training protocols on comprehensive SDM skill development for clinicians are becoming increasingly accessible (75, 76).

Commonly, treatment failure is interpreted as a drug being unsuccessful in inducing disease resolution or eliciting disease control. While this is true, there are other causes and, additionally, varying shades of lack of therapeutic responsiveness which must be recognized in order to preserve the use of an effective or partially effective drug. Firstly, an absolute lack of treatment responsiveness must prompt consideration of either wrong diagnosis, inactive sarcoidosis with high damage burden or medication non-adherence (requiring exploration through SDM). If active sarcoidosis is confirmed, there may be genetic influences on bioavailability of a particular medication that require attention (77, 78). Partial responsiveness, wherein a monotherapy is insufficient to completely quell a disease activity level (that may be of high intensity, or that may outpace the efforts of a particular drug or drug dose) should still be considered valuable. A drug, eliciting partial responsiveness, used in combination with other agents, may provide value in keeping doses of more toxic agents minimal.

Tolerability is another cause of “treatment failure” and may have the greatest potential for salvaging efficacious systemic medication. Ongoing query into patient perceptions of side effects, as can be accomplished with patient self-reported measures, may support earlier interventions that effect tolerability. Being clinically inquisitive in gaining knowledge in the many strands of administration (e.g., route, frequency, dose division, rate of dose escalation, acclimation maneuvers, timing, nutrition, etc.) and palliation (e.g., anti-emetics, anti-diarrheal, etc.) to enhance tolerability, is pivotal (in the context of SDM) to preserving systemic medication use.

Toxicity accounts for another type of “treatment failure.” This area requires dedicated knowledge to preventing and monitoring for toxic medication effects, as well as an opportunity to re-challenge. Often, once an unanticipated toxicity occurs, a person’s confidence in that medication is shaken.

Finally, it is important to note that none of the drugs used in sarcoidosis are “curative” and that relapses occur frequently with treatment interruption, medication holidays or with medication tapers. These relapses should not be interpreted as treatment failure but rather as disease recurrence following early discontinuation of treatment. In cases where a relapse occurs after at first a response was achieved, previously successful therapy should be re-instituted, and a more prolonged treatment course considered (79).

### Ascertaining sarcoidosis vs. other etiology of worsening symptoms

**FIGURE 2:**
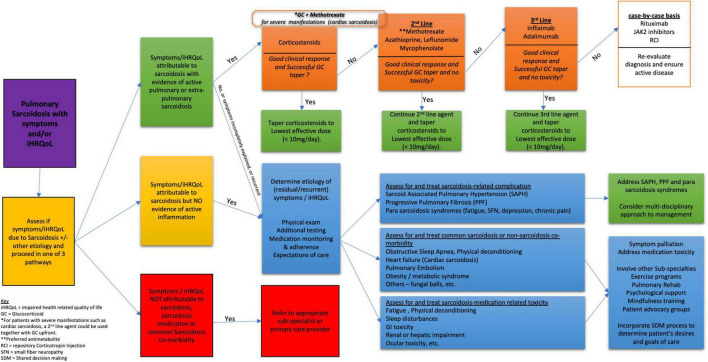
Global approach to treatment of sarcoidosis.

For patients who develop new or worsening symptoms while on therapy, it is critical to consider and evaluate for other causes of these symptoms rather than routinely attributing worsening to failed therapy or to established progressive pulmonary or other organ sarcoidosis manifestation (80, 81). Common sarcoidosis-related complications exhibiting overlapping symptoms requiring consideration include cardiac involvement with either arrhythmia or heart failure, sarcoidosis-associated pulmonary hypertension (SAPH), small fiber neuropathy, and CNS symptoms (80, 82, 83). Fatigue, depression, sleeplessness, physical deconditioning, and obstructive sleep apnea are also very common medication-related, sarcoidosis-related, or non-sarcoidosis-related co-morbidities that may drive the appearance of worsening disease (80–82, 84). Entities contemporaneously seen in sarcoidosis that are crucial to consider are acute or sub-acute bacterial pneumonia, mycobacterial or mycotic infection, cardiovascular disease events common in the general population, and pulmonary embolism or lung and other cancers that have a higher temporal relationship to sarcoidosis than in the general population (85–95). Figure 2 outlines a more global approach to treatment that emphasizes the need for comprehensive patient care (83, 84, 96).

## Challenges affecting drug trials and drug development in sarcoidosis

### Challenges with patient recruitment, patient selection, and cohort enrichment

Careful definition of the target cohort, as much as is possible, is critical in clinical trial design. Lack of attention to patient selection and cohort enrichment could generate data that significantly under-represent the efficacy of a good drug. A central premise of selection is establishing active sarcoidosis in the target organ(s) that will be sufficiently responsive to an efficacious treatment. This is accomplished in either or both of two major ways: (a) demonstration of inflammation consistent with sarcoidosis to the exclusion of other causes, which may be accomplished by positron emission tomography (PET)/computerized tomography (CT) scan in non-neurosarcoidosis, cardiac PET/cardiac magnetic resonance imaging (cMRI) in CS or brain MRI changes suggestive of edema/inflammation in neurosarcoidosis (97–102); (b) demonstration of clinically meaningful progressive disease in target organ(s) that is documented over a defined recent interval of time (for which other causes of worsening have been ruled out). Examples include worsening impairment or extent of disease on imaging, exercise tolerance or pulmonary function tests (PFTs) in lung involvement, worsening left ventricular ejection fraction (LVEF) on echocardiogram or cMRI in CS, or worsening limb strength or balance in CNS involvement. Effective selection also minimizes confounding factors that portend poor likelihood for significant improvement such as concomitant sarcoidosis co-manifestations that are minimally responsive to anti-inflammatory therapy examples of which include presence of severe SAPH in an interstitial lung disease (ILD) trial or extensive fibrosis in patients with pulmonary sarcoidosis. Both these co-manifestations are characterized on PFTs by very low forced vital capacity (FVC) and/or diffusing capacity for carbon monoxide (DLCO).

Additional challenges with patient recruitment include the need to promote diversity and ensure equitable enrollment of minorities and non-white participants. As much as is possible, trial populations should be reflective of the populations affected by the disease of interest. Sarcoidosis occurs three times more commonly in African Americans and presents with disproportionate severity in patients of a lower socioeconomic status (SES) (40, 42, 45, 103, 104), yet most trial populations thus far have not been reflective of this racial prevalence of disease (105, 106). Several studies show that African Americans are less likely to both qualify for and to participate in clinical trials due to several reasons such as mistrust in the system, lack of interest in clinical trials, fear and stigma associated with participation, and a perception that they may not be compliant with trial protocols (107–110). Several strategies that have improved clinical trial participation for minorities and the underserved in other diseases may also be deployed in sarcoidosis (108, 111, 112). These strategies center around the need to address mistrust and misconception, promote increased information and awareness of the benefits of trial participation, and ensure that investigators and study personnel are fully trained in cultural and racial competencies (108, 112). Ensuring feasibility studies are undertaken prior to commencement of large trials and involving patients in the design stages of trials will also help to address several of these challenges (113).

Studies focused on non-pulmonary manifestations of sarcoidosis face the unique challenge that very few patients (< 2% in one large cohort) (3) will have isolated non-pulmonary manifestations of sarcoidosis. Consequently, trial designs in pulmonary sarcoidosis may need to be adapted to enrich for other organ manifestations of disease without detracting from pulmonary endpoints, or conversely, to focus on a very small subset of patients with very clear-cut endpoints—such as focusing on chronic cutaneous sarcoidosis and using the sarcoidosis activity and severity index (SASI) (114, 115) as an endpoint or focusing on optic neuritis rather than all neurosarcoidosis. To this end, leveraging ongoing studies in biomarker, proteomics, and metabolomics research will be crucial to guide patient selections that enrich trial populations with patients that have active disease in multiple non-competing organs (116, 117). Routine use of PET scans to detect evidence of active disease in the lungs and extra-pulmonary organs, may also begin to address this issue (62, 118–122).

Besides RCTs, the use of large global registries with well phenotyped patients is also a critical step to systematically study various forms of high-risk or severe manifestations of sarcoidosis and their optimal treatment regimens. Sarcoidosis is a rare disease with heterogenous manifestations, and a potential marginal benefit of registries compared with RCTs is the ability to include a more heterogenous (and more representative) sarcoidosis population so as to gain large numbers and gain insight into the use of off-label therapeutics and novel therapeutic approaches for uncommon and severe disease manifestations. Several such registries are currently in existence and may begin to yield some much-needed information in this regard (14, 123–125).

### Identifying appropriate endpoints

Another limitation of drug development in sarcoidosis has to do with identifying validated measures that reflect clinically meaningful response to therapy. Identifying and validating outcome measures is one of the greatest tasks at hand in organ-specific or multi-organ trial design in sarcoidosis. Even when considering a prevalent manifestation such as pulmonary sarcoidosis, the most used measures such as FVC can be flawed in capturing clinically meaningful change (126); and may miss a large subset of patients with ventilatory defects affecting other PFT parameters (127). Another example is though improvement in HRQoL along with the minimum clinically important difference (MCID) for various HRQoL instruments has been reported with some treatment regimens (128, 129), correlative changes in HRQoL to other endpoints such as physiologic function or steroid-tapering are still lacking.

The selection of outcome measures is predicated upon which outcomes or endpoints (whether primary, secondary, or exploratory) are deemed important in validating the hypothesis while remaining cognizant of the drug’s mechanism of action and anticipated side effects. For example, in clinical trials targeting lung involvement and measuring changes in physiologic function as primary outcome, FVC, FEV1 or DLCO is often selected while changes in physical function may warrant use of 6-min walk distance and other outcome measures assessing dyspnea, cough or HRQoL may be selected from a variety of PROMs intended to measure each of these as secondary or exploratory outcomes. As much as is possible, outcome measures should be authenticated to demonstrate content validity, reliability, discrimination between similar but different situations (e.g., SAPH vs. sarcoidosis-ILD, respiratory decline vs. anxiety), and responsiveness to changes over time that correlate with clinically meaningful change in disease state. Measures are more likely to be successful when they demonstrate high precision, easy interpretability, cost effectiveness, accessibility, and are without undue risk regarding patient safety, comfort, and fatigability. Measures that are easy and logical to complete and that provide immediate real time feedback to patients are also more likely to be accepted.

As noted, the ERS guidelines stress the two major reasons for treatment: avoid organ loss or death (danger) and/or improve HRQoL. Unfortunately, none of currently available literature has used these as a specific endpoint. Since mortality from sarcoidosis, death alone as an endpoint has not been a practical primary endpoint, the time to clinical worsening (TTCW) which includes a composite of predefined endpoints such as disease-related hospitalization, death, transplantation, or worsening of 6-min walk or FVC of 5–10% have been used as primary endpoints in trials for SAPH and pulmonary fibrosis (130, 131). However, most patients entering trials still have enough reversible disease making hospitalization, death, or lung transplantation far less likely. Thus, these endpoints may not be sensitive in a treatment trial for anti-inflammatory therapy.

Improving HRQoL should be a core outcome of all clinical trials in sarcoidosis (5). Unfortunately, the use of diverse PROMs makes comparison across studies difficult. There is a need to identify core sets of outcome measures for organ-specific and systemic sarcoidosis, respectively. The recently convened Sarcoidosis Clinical Outcomes Task Force (SCOUT) has identified several commonly reported outcome measures in pulmonary sarcoidosis with a view to develop a set of core outcome measures that can be uniformly applied across studies in pulmonary sarcoidosis (22).

Another endpoint accepted as reasonable is the ability to taper steroid dosage (steroid-sparing) (22). While reduction of steroids is clinically meaningful and important to patients, subjects enrolled in the placebo arm of placebo-controlled studies, are expected to be unable to achieve steroid reduction, thus remaining on moderate steroid doses for prolonged periods. This is problematic for a few reasons. The symptoms of prolonged steroid use can be intolerable to patients especially as patients are increasingly aware of steroid-sparing treatment alternatives, thus creating a vulnerability to patient retention. While drop-out in the placebo arm may appear to be data in favor of treatment, unless drop-out is a primary, or at least secondary, endpoint this information will not be captured as a meaningful outcome. Another is an ethical concern considering the availability of steroid-sparing agents and the well-known short and long-term toxicities of prolonged steroid use. Further, as sarcoidosis clinical trials become more plentiful, investigators are going to select among the studies they feel are optimal for their patients’ health and safety and steroid withdrawal studies are unlikely to be preferred among other available studies. Other concerns for the use of steroid-sparing as an endpoint exist. Sarcoidosis is a multi-organ disease; therefore, the target organ may not be the organ that relapses when steroids are withdrawn thus creating conflicting trial results. For example, a patient in a pulmonary sarcoidosis trial may develop new or worsening uveitis as prednisone is withdrawn, while lung function remains stable. This information needs to be captured in trials. Using a customized approach to TTCW as an endpoint will capture these adverse events. Next, patients on medium to high dose steroids may develop steroid withdrawal symptoms unrelated to the efficacy of the trial drug and these may confound trial results. These concerns notwithstanding, several studies in pulmonary sarcoidosis have shown statistically significant steroid-sparing (132, 133) and it remains an important outcome to patients dealing with sarcoidosis and the toxic effects of steroids (5, 21, 22). Measures to limit the extent of steroid toxicities or to pro-actively manage their onset may be necessary for patients on placebo who require escalating steroid doses (28).

A possibly more efficient and patient-centered strategy in non-neurosarcoidosis might be the use of changing PET/CT values over time. The use of PET/CT is likely to shorten trial length, confer greater precision of change (126), allow for the flexibility of enrolling patients with clinically active disease whether treatment naïve, on corticosteroids or a steroid-sparing agent and confer the ability to use the addition or tapering of non-study drug as an outcome. Although very attractive as an end point, apart from CS, changes in PET/CT scans have not yet been validated as outcome measures in sarcoidosis and controversies exist as to whether SUVmean or SUVmax should be used (98, 122, 134). Further, changes in PET/CT imaging have not always correlated to changes in clinical parameters (56, 98, 122, 134–138). Nonetheless, use of change in PET/CT values over time remains a very promising endpoint and work on establishing its role in this regard remains ongoing.

Hierarchical composite endpoints (HCE) whereby multiple relevant outcomes or components ranked in order of clinical importance/relevance and combined into a single ordinal outcome may also be considered. These components would have to be adapted to both the organ of interest and the study drug under investigation and should capture both the most favorable and least desirable aspects of a drug or intervention (139, 140). For example, a trial evaluating the role of a new molecule in pulmonary sarcoidosis, may wish to evaluate its role as a steroid-sparing agent while concomitantly assessing its toxicity profile, and effect on HRQoL and FVC. Such a study may wish to prioritize steroid-sparing and toxicity profile or HRQoL over FVC and may consequently design a HCE wherein the highest (best possible) rank is given to patients who are able to taper steroids to < 10 mg/day of prednisone or prednisone equivalent, have no reported toxicity, experience a clinically significant improvement in HRQoL, and have a prespecified improvement in FVC. Patients who are unable to taper steroids, but who otherwise meet all the other criteria may be given a second rank and so on and so forth—with the worst rank given to patients who do not meet any of the prescribed criteria. The study would then identify and report what proportion of patients are able to achieve a certain rank or higher as an outcome. HCE have not been used in sarcoidosis trials, however, several non-sarcoidosis trials suggest that they provide a sensitive endpoint to detect treatment effect with smaller sample sizes and in shorter time periods (139).

Table 2 lists the various potential endpoints of clinical trials in sarcoidosis.

**TABLE 2:** Proposed endpoints for clinical trials in sarcoidosis (5, 21, 22).

Organ involvement	Domain	Measure	Comments
Pulmonary sarcoidosis	**Symptoms	Dyspnea—mMRC, BDI/TDI Cough—Leicester scale Fatigue—FAS	This should be customized to capture multi-organ and/or extra pulmonary involvement.
	*Physician judgment	Clinical judgment of improvement, worsening or progression.	This is applicable to systemic and all organ-specific forms of sarcoidosis.
	*Steroid sparing	% Reduction in steroid dose, Cumulative steroid dose, Duration of time at minimal steroid doses, % Of participants able to achieve steroid taper to < 10 mg/day.	Consider analyzing drop-out from placebo arm as a secondary outcome. Confounding results may occur from withdrawal from steroid or flare-ups in non-target organs. Measures of steroid toxicity and ways of addressing them need to be put in place.
	Radiology/evidence of activity	Changes in PET/CT chest imaging	Changes in PET scans will need to be defined in terms of SUVmean or SUVmax. There is a need to determine what constitutes a meaningful difference in SUV levels.
	*Medication toxicity/tolerance	Serious AEs, Life threatening AEs, AEs leading to discontinuation of therapy Other AEs	This should be captured in all clinical trials and tailored to investigational drugs and organ system targeted.
	Pulmonary function	FEV1, FVC, DLCo, CPI	There is a need to determine what is clinically meaningful disease specific change in FVC, FEV1 and DLCo for patients with pulmonary sarcoidosis. The CPI has also been validated as a prognostic severity marker in pulmonary sarcoidosis.
	Exercise capacity	6MWD	There is a need to determine what constitutes meaningful change in 6MWD for patients with pulmonary sarcoidosis.
	*HRQoL	SGRQ, SF-36, SAT-Lung FAS KSQ General Health; KSQ Lung	Various PROMs have been used to capture HRQoL. There is a need to create core sets of outcome measures for organ specific and systemic sarcoidosis.
	Mortality	Mortality often not feasible Consider composite outcome—TTCW	TTCW is a predefined composite endpoint that can be customized to capture such events as disease-related hospitalization, all-cause hospitalization, death, transplantation, worsening of 6MWD, PFT or symptom burden.
Cutaneous Sarcoidosis	Cutaneous sarcoidosis disease activity HRQoL	PGA, SASI, CSAMI, Photographs SAT skin, KSQ Dermatology Questionnaire, SAT Fatigue	
Cardiac Sarcoidosis	Symptoms Radiology/Evidence of Disease Activity Exercise Capacity Mortality	Arrythmias/arrythmia burden cPET Scan, cMRI, Echocardiogram (LVEF) 6MWD Mortality is often not feasible. Consider composite outcomes assessing all-cause hospitalization, cardiac hospitalization,	Note that mortality will likely never be feasible in view of rarity of disease and much improved prognosis. Though composite outcomes are more achievable, sample size is likely to be prohibitive in view of rarity of disease and much improved prognosis.
Neurosarcoidosis	Imaging/evidence of disease activity HRQoL	MRI Measures assessing cognitive functioning, Functional independence, strength measures of limbs, General Health status questionnaires.	
Others Ocular Renal Hypercalcemia	HRQoL measures	General and organ specific HRQoL measures	This can be customized for each organ involved.

**Should be customized to reflect the specific organ(s) of interest.

*Applicable to all organ manifestations of disease.

HRQoL, health related quality of life; TTCW, time to clinical worsening; PFT, pulmonary function tests, FVC, forced vital capacity; FEV1, forced expiratory volume in 1 second; DLCo, diffusion capacity for carbon monoxide; SGRQ, Sant Georges Respiratory Questionnaire; SF-36, Short form-36; SAT-Lung, sarcoidosis assessment test lung component; FAS, fatigue assessment score; KSQ, Kings sarcoidosis questionnaire; mMRC, modified Medical Research Council; BDI/TDI, baseline dyspnea index/Transitional dyspnea index; PET/CT, positron emission tomography/computed tomography scan; cPET, cardiac PET scan; SUV, standardized uptake Value; MRI, magnetic resonance imaging; cMRI, cardiac MRI; AE, Adverse event; 6MWD, 6-min walk distance; PGA, Physician Global Assessment; SASI, sarcoidosis activity and severity instrument; CSAMI, cutaneous sarcoidosis activity and morphology instrument; LVEF, left ventricular Ejection Fraction; CPI, composite physiological index; a weighted index of pulmonary function variables.

### Subject retention

Subject retention relies on principles of patient safety, comfort, anticipating medication tolerability and cost of participation. This necessitates incorporation of preventive measures and management of toxicities related to steroid-tapering studies. Such measures include protocols to ensure gastric, bone, endocrine and cardiovascular protection as well as measures to address psychiatric and sleep disturbance that follows any available published guidelines for prolonged steroid use. On the other hand, investigator protocols to help manage side effects of study drug that are anticipated to be frequent and decrease study drug tolerability, should be carefully developed with supportive communication aids and patient brochures in the event side effects arise.

### Fair reimbursement for participation

Patients are the most valuable element in clinical trials, and it is their participation that enables science and treatment development to advance. Most patients are motivated to participate in trials. However, “motivation” to benefit self or others is not enough for a patient to be able to participate in a clinical trial, as participation in clinical trials often requires a level of financial stability and job security that allows compliance with complex trial schedules and prolonged time off work for multiple study visits—sometimes at centers far away from home. Consequently, only patients that can afford the financial and other losses associated with clinical trials are able to participate without the support of financial coverage for expenses and collateral costs of participation (141, 142). For patients diagnosed with sarcoidosis—who have been shown to experience significantly less earnings, higher work-lost days, greater risk of job loss, and greater difficulty qualifying for income support for lost wages due to disease (42, 143, 144)—participation in clinical trials may present a significant socio-economic burden that few are equipped to bear.

Patient costs of research trial participation can be divided into two broad categories. D*irect expenses* paid by patients to attend visits (travel, meals for long visits) and *collateral costs* to the patient (time off work, the stress of negotiating time off from work, time recording in and dealing with technical issues related to e-diaries, potential electricity for charging, and data use for e-diaries). A potential third category might be *procedural burden* which might relate to the complexity and potential discomfort of study procedures. While a fourth category might be *probability of benefit* which relates to foregoing other available treatment to participate in a placebo trial or a trial of a medication without clinical precedent, or as is common in sarcoidosis to remain on prolonged toxic medications such as prednisone. Participation in clinical trials occurs on top of the usual anticipated annual financial losses for patients and family members related to the disease itself (28, 42, 143, 145). Subjects will continue to need to take sick days and vacation days or lose wages for comprehensive care of their health status that is not supplied within the context of the trial. Supporting fair reimbursement for clinical trial participation protects diversity and inclusion of non-white and lower SES participants who could not afford the financial loss associated with RCT participation (141, 142, 146). The validity of coercional incentive related to reimbursement is rapidly losing ground and recent studies supports the position that payment to economically vulnerable populations is ethically justified and indeed desirable when certain conditions are met (141, 142, 147–149).

### Patient research partners

Some of the most valuable guidance on feasibility, subject retention, and natural history of disease come from patients themselves. Patients are experts in their disease and have an idea of what other patients are willing to tolerate and make trade-offs for. For e.g., the likelihood that patients will remain on stable dose of steroids if study drug shows no efficacy, or the degree of imposition of a daily e-diary. Patient research partners (PRPs) are now accepted as an important element of successful clinical trial design (150). Inclusion of PRPs from the inception of a study imparts expertise on foundational aspects of trial success such as feasibility and subject retention (150, 151). PRPS provide their general expertise which leverage advantages in brainstorming solutions and offering insight from their unique lens on disease behavior (152). There is work to be done on remuneration for PRP effort and their involvement through to publication of results with appropriate acknowledgments, but that is beyond the scope of this manuscript (153, 154).

## The immunopathogenesis of sarcoidosis as it influences therapeutic drug trials

**FIGURE 3:**
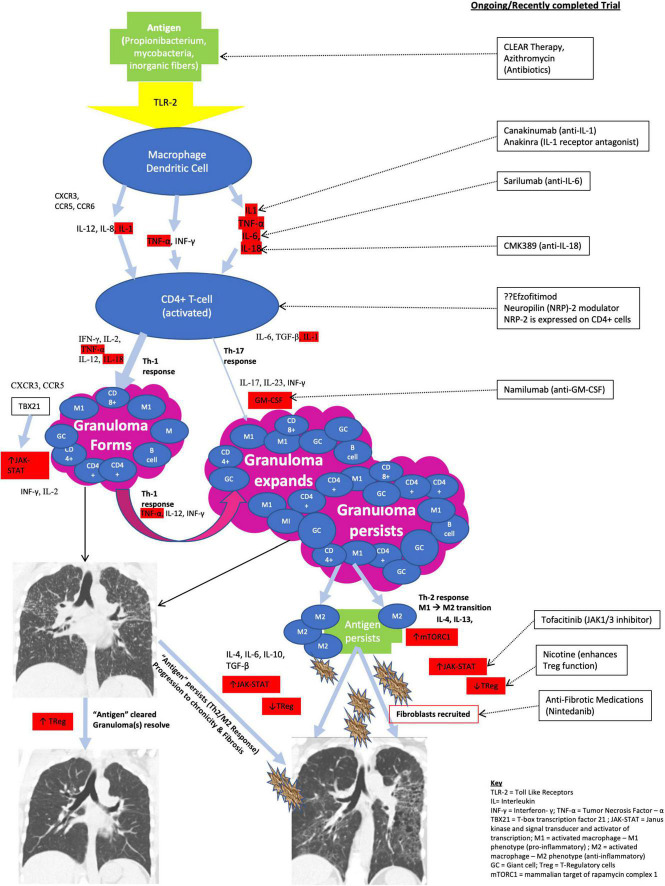
Immunologic cascade resulting in sarcoid granuloma formation.

The sarcoid granuloma is the immunohistopathologic hallmark of sarcoidosis (1, 96, 155). It has been shown to result from an aberrant CD4 + Th1/Th17 cell mediated immune response to a yet unknown (presumptive) environmental or occupational exposure/stimulus in a genetically predisposed individual (96, 156–160). The immunologic cascade resulting in sarcoid granuloma formation has been described and is outlined in Figure 3. It may broadly be categorized into three main phases: granuloma formation, granuloma propagation/expansion, and persistence of granuloma associated with chronic disease and progression to fibrosis (96). Not every patient progresses through all the three stages to fibrosis. Most patients will have resolution or stabilization of their disease, and this can occur at any stage (96, 157). Only 10–30% of patients will develop a chronic non-remitting disease with progression to fibrosis (8, 161, 162). Certain HLA subtypes and African American ancestry have been associated with chronic progressive fibrotic disease (156, 163).

As noted above, the inciting “antigenic” stimulus in sarcoidosis is unknown, however, several studies support that an inhaled infectious, organic, or inorganic antigen acquired from various occupational and environmental exposures such as moldy environments, metalworking, firefighting, agricultural employment, and occupational exposures to insecticides and building supplies is implicated in disease etiology (96, 156, 164). A double hit theory either from the same “antigen” with a prolonged intervening latent period, or multiple insults from various synergistic “antigens” has been proposed (96, 165). The inciting antigen may arise from one or more sources, such as Propionibacteria, mycobacteria, or inorganic fibers. A dysregulated immune response to antigenic stimulus (or stimuli) from mycobacterial catalase-peroxidase G (mKatG), the 6-KDa early secreted mycobacterial antigenic protein (ESAT-6), and Propionibacterium nucleic acids has been associated with a granulomatous condition (166–175). A possible role for serum amyloid A (SAA) inappropriately accumulated in response to mycobacterial infection/exposure, or another unknown stimulus has also been suggested (176, 177). Although possible exposure to mycobacterial proteins remains one of the most plausible etiologic risk factors for sarcoidosis, a recently published large RCT did not show any clinical or physiologic response to anti-mycobacterial therapy in patients with pulmonary sarcoidosis despite a significant reduction in ESAT-6 levels (106); and several studies have failed to culture mycobacteria from sarcoidosis tissues (178, 179).

The inflammatory response in sarcoidosis is initiated by the innate immune system through activation of membrane-bound pattern recognition receptors (mbPRRs) on the surface of antigen presenting cells (macrophages and dendritic cells) at the alveolar epithelial surface (168). These mbPRR include toll-like receptors (TLR), RIG-I-like receptors, and nucleotide-binding oligomerization domain and leucine-rich repeat-containing receptors (NLRs) which constitute one component of the NLRP inflammasome (168, 180). Activation of alveolar macrophages (*via* TLR) results in activation of effector proteins (such as caspase-1) and cleavage of inactive forms of interleukin (IL)-1β and IL-18 into their active forms (168, 180). Activated alveolar macrophages produce large amounts of several proinflammatory and Th1-skewing cytokines such as IL-1, IL-6, IL-8, IL-12, IL-18, interferon (IFN)-γ, and TNF-α (96, 157, 168). These cytokines in conjunction with several chemokines and chemokine receptors (CXCR3, CCR5, CCR6) also released by activated macrophages activate the adaptive immune system and upregulate the process of granuloma formation and expansion through activated CD4 + and CD8 + T-cells (96, 157, 168).

The core of the sarcoid granuloma consists predominantly of activated CD4 + T helper (CD4 + Th) lymphocytes with rare scattered CD8 + T cells and B cells in the periphery (157). The adaptive immune response in sarcoidosis is a predominant CD4 + type 1 helper (CD4 + Th1) response (181). Activated alveolar CD4 + T-lymphocytes produce high levels of IFN-γ, IL-2, and TNF-α as well as high levels of IL-12 and 1L-18 which skew the immune response toward a Th1 pathway and cause increased expression of Th1-associated chemokines CXCR3 and CCR5 which amplify the Th1-oriented response (96, 157, 182, 183). The activated CD4 + Th1 cells also upregulate the Th1-specific transcription factor (T-box transcription factor 21 [TBX21]) which promotes further differentiation of CD4 + Th cells down the Th1 pathway (96, 184). TBX21 activates/regulates the Janus kinase and signal transducer and activator of transcription (JAK-STAT) pathway and controls the Th1 hallmark cytokine IFN-γ (96). The JAK-STAT pathway has been proposed as a potential drug target in sarcoidosis (18, 185, 186).

Alveolar CD4 + Th lymphocytes may also differentiate down a Th17/Th17.1 effector pathway under the influence of IL-1, IL-6, and TGF-β (157, 187–189). Th17 cells produce IL-17 and INF-γ, and their survival and proliferation is dependent on IL-23 (187, 190, 191) which regulates the process of Th17 cell differentiation (157, 160, 191–193) and has also been shown in some cases to initiate a more proinflammatory process (resulting in persistence of the sarcoid granuloma) through the production of granulocyte-macrophage colony stimulating factor (GM-CSF) (96, 194–196). The Th17/17.1 pathway is less frequently employed in sarcoidosis but has been implicated in the development of chronic progressive disease (156, 188, 197). A large phase II multi-center randomized trial that evaluated the role of Ustekinumab (a fully human IgG1 monoclonal antibody directed against IL-12/IL-23) in patients with chronic pulmonary and cutaneous sarcoidosis refractory to corticosteroids found that there was no significant difference in pulmonary function, health related quality of life (HRQoL) or skin assessment score in patients on Ustekinumab vs. placebo after 6-months of therapy (105). A drug trial directed against GM-CSF in patients with chronic refractory pulmonary and CS is ongoing and will be discussed further below (198, 199).

Failure to clear the inciting antigen and persistence of a dysregulated immune response has been associated with the development of chronic disease and progression to fibrosis in sarcoidosis (200). Activated macrophages drive the inflammatory process associated with granuloma formation. In the classic antigenic model, phagocytic clearance of the offending pathogen results in resolution of the inflammation and the granuloma, however, persistence of the antigenic stimulus results in ongoing inflammation and propagation of the sarcoid granuloma (201). Alveolar macrophages may be classified as M1 or M2 depending on the cytokine microenvironment (168). MI macrophages are activated by IFN-γ and produce proinflammatory cytokines (TNF-α and IL-12) whereas M2 macrophages are generated in the presence of Th2 cytokines (IL-4 and IL-13) and produce immunosuppressive, immunoregulatory, anti-inflammatory and profibrotic cytokines (168). IL-4, IL-6, IL-10, and TGF-β are anti-inflammatory cytokines that inhibit IL-2 and INF-γ and facilitate fibroblastic recruitment leading to extracellular matrix deposition and fibrosis (202, 203). It is thought that transition from a Th1/M1 predominant pro-inflammatory cytokine response to a Th2/M2 anti-inflammatory cytokine response promotes persistence of the sarcoid granuloma and development of chronic disease (96, 202, 203). IL-13 promotes the differentiation of M1 to M2 macrophages and has also been shown to activate the metabolic check point kinase mammalian target of rapamycin complex 1 (mTORC1) (96, 204). mTORC1 has been implicated in granuloma formation through its role in activating macrophages and promoting their differentiation into epithelioid cells and multinucleated giant cells (96, 185, 204). Impaired autophagy resulting from excessive stimulation of mTORC1 pathway has been implicated in the failure to eliminate antigens and shown to contribute to granuloma persistence and chronicity (96, 204, 205).

Other immune mechanisms associated with persistence of the sarcoid granuloma and thus of the development of chronic progressive fibrotic disease include activation of the JAK-STAT pathway (185, 186) and impaired immunosuppressive function of T-regulatory cells (Tregs) (206–208). Studies show that sarcoid derived Tregs fail to inhibit production of TNF-α, INF-γ, and IL-2 all of which contribute to granuloma growth and expansion (206, 207, 209).

## Ongoing, future, and recently concluded clinical drug trials in sarcoidosis

ClinicalTrials.gov is a publicly available database of all privately and publicly funded clinical trials conducted around the world. It includes all studies conducted within the 50 states and in over 200 countries including Japan and Europe (210).

**FIGURE 4:**
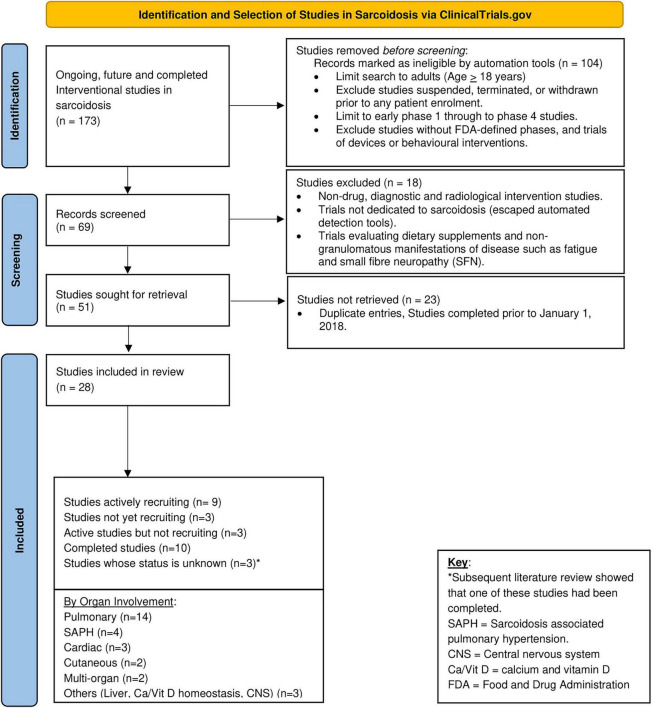
Active, future, and recently concluded clinical trials in sarcoidosis in the past 5-years (2018–2022).

A search on ClinicalTrials.gov of all ongoing, future, and completed interventional clinical trials in sarcoidosis yielded 173 results. Limiting the search to adults (age 18 and over), drug intervention, and early Phase 1 through Phase 4 trials, as well as excluding trials of devices or behavioral interventions, and studies that have been withdrawn, suspended, or terminated resulted in 69 studies. During manual review of these studies, duplicate entries as well as trials evaluating nutritional supplements, non-granulomatous manifestations of sarcoidosis, and diagnostic, radiologic, and non-drug interventions were excluded. We also excluded trials completed before 2018. Trials evaluating the same molecule for different disease manifestations (such as for pulmonary and CS) or where an early phase trial has been completed and late phase trial initiated were acknowledged as independent studies. Twenty-eight trials were identified. Fourteen of these trials are in pulmonary sarcoidosis, four in SAPH and three in CS. There are only two trials each in cutaneous and multi-organ sarcoidosis and one each in hepatic, CNS sarcoidosis and sarcoidosis affecting the calcium and Vitamin D homeostatic balance (Table 3 and Figure 4). As at the time of publication, nine of these trials are actively recruiting, three are anticipated to start recruiting and another three are reported as active but not recruiting. Three of the studies are reported to have an unknown status, however, on further literature review, one of these studies has been completed with results published (131). An additional ten studies are reported as completed; six of these have preliminary results yet only three of these study results have been published in peer-reviewed journals (106, 128, 211) and one in abstract form (132). Of the 15 active and/or anticipated studies, 11 are evaluating drug molecules and four are evaluating alternative treatment approaches using already established drugs as listed in Table 1. Three of the active drug intervention trials are each in pulmonary sarcoidosis and SAPH (27% each), two are in CS (18%) and there is only one ongoing study in each of hepatic, calcium/Vit D homeostatic imbalance and multi-organ disease. Figure 4 outlines the search process used to identify trials included in this manuscript and Table 3 provides a summary of the active, future and recently completed trials in sarcoidosis.

**TABLE 3:** Summary of ongoing, anticipated and recently completed studies in sarcoidosis from 2018 to 2022.

Organ system	Study title	NCT number	Study status	Sample size/ completion date	Primary outcome (s)	Secondary outcome measure(s)
Pulmonary sarcoidosis	Efficacy and safety of IV Efzofitimod in Patients with Pulmonary Sarcoidosis	NCT05415137 (Phase 3) NCT03824392 Phase 2/completed	Planned but not started	#264/January 2025 #37/July 2021	Steroid tapering at 48 weeks Safety and Tolerability	Change in FVC Change in KSQ-Lung score Steroid tapering, cumulative steroid dose, Immunogenicity.
	RCT of Hydroxychloroquine Combined with Low-dose Corticosteroid in Pulmonary Sarcoidosis. (QUIDOSE)	NCT05247554 (Phase 3)	Planned but not started	#200/March 2024	Change in FVC at 26 weeks	Not stated
	Efficacy and Safety of SQ Namilumab in Participants with Chronic Pulmonary Sarcoidosis (RESOLVE-Lung)	NCT05314517 (Phase 2)	Actively recruiting	#100/January 2025	Change in FVC at 26 weeks	Steroid sparing, Safety and Tolerability, Change in PROs (not specified) Cumulative steroid dose and toxicity Change in SASI and ePOST, Change in HRCT and PET imaging, Change in 6MWD
	Efficacy, Safety and Tolerability of IV CMK389 in Patients with Chronic Pulmonary Sarcoidosis	NCT04064242 (Phase 2)	Actively recruiting	#66/July 2023	Change in FVC at 16 weeks	Steroid tapering, Composite index of change in FVC & 6MWD, Change in FEV1, 6MWD Change in PET imaging
	Effectiveness of Methotrexate vs. Prednisolone as First-line Therapy for Pulmonary Sarcoidosis (PREDMETH)	NCT04314193 (Phase 4)	Actively recruiting	#138/January 2025	Change in FVC at 24 weeks	Time to pulmonary (FCV) improvement Change in DLCO Change in Biomarkers (sACE, sIL-2R, T-cell biomarkers). Change in KSQ (all domains), CRQ, GRC, EuroQol, FAS Change in mMRC Medication tolerance and change in PESaM
	Efficacy and Safety of Two Glucocorticoid Regimens in the Treatment of Sarcoidosis (SARCORT)	NCT03265405 (Phase 4)	Actively recruiting	#86/June 2022	Relapse or Treatment failure at 18-months	Time to relapse or treatment failure, Proportion of patients with response to therapy, change in FVC, cumulative prednisone dose, prednisone toxicity, HRQoL (SHQ and FAS)
	Phase II Investigation of Antimycobacterial Therapy on Progressive, Pulmonary Sarcoidosis	NCT02024555 (Phase 2)	Completed	#97/April 2019	Change in FVC at week 16	Radiographic improvement (CXR) Change in 6MWD, Dyspnea, Change in FAS, SGRQ, KSQ, Adverse events Change in FEV1
	Nicotine Treatment for Pulmonary Sarcoidosis: A Clinical Trial Pilot Study	NCT02265874 (Phase II)	Completed	#57/Nov 2021	Change in FVC	Change in CT imaging
	Azithromycin a Treatment for Pulmonary Sarcoidosis CAPS	NCT04020380 (Phase 2)	Completed	#21/June 2020	Change in cough count at 12 weeks	Change in severity of and urge to cough Change in Leicester cough questionnaire Change in KSQ total score
	Tofacitinib Hypothesis-generating, Pilot Study for Corticosteroid-Dependent Sarcoidosis	NCT03793439 (Phase 1)	Completed	#5/June 2021	Steroid Sparing (50% reduction in CS requirement) at week 16	Change in STAT1 mediated Genes by peripheral blood RNA sequencing
	ActharGel in Participants with Pulmonary Sarcoidosis (PULSAR)	NCT03320070 (Phase 4)	Completed	#55/November 2021	Change in FVC and DLCO at week 24; change in HRCT	Change in FAS, steroid taper
	Study of efficacy, safety, and tolerability of ACZ885 (Canakinumab) in Patients with Pulmonary Sarcoidosis	NCT02888080 (Phase 2)	Completed	#40/March 2019	Change in FVC at week 24	Change in PET/CT, HRCT Change in 6MWD Change in FEV1, DLCO
Fibrotic pulmonary sarcoidosis (FPS)	Pirfenidone for Progressive Fibrotic Sarcoidosis. (PirFS)	NCT03260556 (Phase 4)	Completed	#60/March 2020	Time To Clinical Worsening (TTCW)	Change in FVC and Composite physiologic Index
SAPH	Safety and efficacy of oral selexipag in participants with SAPH (SPHINX)	NCT03942211 (Phase 2)	Actively recruiting	#74/September 2024	Pulmonary Vascular Resistance (PVR) week 26	Not stated
	Inhaled Treprostinil in patients with SAPH (SAPPHIRE)	NCT03814317 (Phase 2)	Actively recruiting	#10/October 2022	PVR at week 16 Mean Pulmonary artery pressure (mPAP) at week 16	Change in 6MWD Change in FEV1 and FVC Change in cMRI Change in BNP and WHO functional class
	A dose escalation study to assess the safety and efficacy of pulsed inhaled nitric oxide in subjects with pulmonary hypertension associated with pulmonary fibrosis or sarcoidosis on long term oxygen therapy.	NCT03727451 (Phase 2)	Active, not recruiting	#17/March 2022	mPAP PVR, Pulmonary capillary wedge pressure (PCWP), cardiac output (CO) and change in 6MWD at week 16	Safety and tolerability Distance saturation product, Dyspnea HRQoL using St. Georges Questionnaire
	Riociguat for Sarcoidosis Associated Pulmonary Hypertension (RioSAPH)	NCT02625558 (Phase 4)	Status unknown	#60/Oct 2018	TTCW	Adverse Events, Change in FVC, HRQoL (instrument not specified), 6MWD
Cardiac sarcoidosis	A study to assess the safety, tolerability, and efficacy of SQ Namilumab in Participants with Active Cardiac Sarcoidosis. (RESOLVE-Heart)	NCT05351554	Planned but not started	#30/January 2024	Safety and tolerability (Incidence of adverse events)	Change in cPET imaging, arrhythmia burden and echocardiogram findings. Hospitalization for cardiac events. Cumulative steroid dose and toxicity. Change in FAS and subject Global assessment
	Interleukin-1 Blockade (daily SQ Anakinra for 4 weeks) for Treatment of Cardiac Sarcoidosis (MaGiC-ART)	NCT04017936 (Phase 2)	Actively recruiting	#28/December 2023	Change in C-reactive protein at 28-days	Change in cPET and cMRI. Serious cardiac events (summation of hospitalizations and death due to cardiac causes)
	Cardiac Sarcoidosis Randomized Trial (CHASM-CS-RCT)	NCT03593759 (Phase 3)	Actively recruiting	#194/December 2024	Change in perfusion and rest scores on cPET scan	Mortality, Cardiovascular hospitalizations, medication related adverse events, GC toxicity, medication compliance, BMI and HRQoL (KSQ, SF 36, SAT), ventricular arrhythmia burden, complete heart block, echocardiography
Cutaneous Sarcoidosis	Open-label Trial of Tofacitinib in Cutaneous Sarcoidosis and Granuloma Annulare	NCT03910543 (Phase 1)	Completed	#15/June 2021	Change in CSASI at 26 weeks	Change in Skindex, Change in PET-CT
	A Clinical Study of Tranilast in the Treatment of Sarcoidosis	NCT03528070 (Early Phase 1)	Status unknown	#56/December 2020	Change in size of skin lesion Change in FVC at 12-months	Not stated
Multi-Organ Sarcoidosis	Sarilumab in Patients with Glucocorticoid-Dependent Sarcoidosis	NCT04008069 (Phase 2)	Active, not recruiting	#15/July 2027	Flare-free survival at 2-weeks	Change in ePOST score, FACIT-F, SASI, 68/66 Joint evaluation, Steroid sparing. change in FVC, FEV1, change in liver and renal function
	Efficacy of Remission-induction Regimen with Infliximab for Severe Extrathoracic Sarcoidosis (EFIRTES)	NCT03704610 (Phase 3)	Completed	#31/September 2021	Change in ePOST score at week 6	Change in ePOST score at week 22
Others	Vitamin D Homeostasis in Sarcoidosis Ursodeoxycholic Acid (UDCA) for Hepatic Sarcoidosis CNS Sarcoidosis and Acthar Gel	NCT03621553 (Phase 4) NCT03602976 (Phase 2) NCT02298491 (Phase 4)	Active, not recruiting Completed Completed	#90/December 2023 #10/July 2023 #4/Nov 2020) (completed)	Change in Lung Function at week 24 Reduction in ALP and GGT Total number of lesions assessed at 1 year	Change in KSQ, 6MWD Change in serum CBC, Vit D, sACE, CRP and several other biomarkers Change in PET/CT and bone density scores Not stated. HRQoL (Treatment satisfaction QoL) measures, change in PDDS, MoCA, SF-36 and Beck depression Inventory-11

CRQ, Chronic Respiratory Questionnaire; KSQ, Kings Sarcoidosis Questionnaire; GRC, Global rating of change Scale; PESaM, Patient Experience and Satisfaction with Medication Questionnaire; FAS, Fatigue Assessment Scale; PET, positron tomography emission scanning; Cpet, cardiac PET scan; cMRI, cardiac magnetic resonance imaging scan; LGE, late gadolinium enhancement; ePOST, extrapulmonary physician organ severity tool; SASI, Sarcoidosis Activity and Severity Index; CSASI, Cutaneous Sarcoidosis Activity Index; FVC, Forced Vital Capacity; FEV1, Forced Expiratory Volume in 1 s; SKindex, skin-related quality of life metric; BNP, Brain Natriuretic Peptide; WHO, World Health Organization; CS, Corticosteroid; ALP, Alkaline phosphate; GGT, gamma glutamyl transferase; SAT, Sarcoidosis Assessment Tool; PDDS, Patient Determined Disease Steps; MoCA, Montreal Cognitive Assessment.

Select studies evaluating novel therapeutic agents and alternative therapeutic regimens are further discussed below. Studies in SAPH are not discussed further as they are considered outside the scope of this manuscript.

## Studies in pulmonary sarcoidosis

Most of the studies in pulmonary sarcoidosis are evaluating new molecules and two are evaluating alternative treatment approaches aimed at minimizing steroid exposure. Two recently completed studies with divergent results provide excellent learning opportunities and are reviewed.

### Studies of novel therapeutic agents in pulmonary sarcoidosis

#### Study of the efficacy, safety and tolerability of CMK389 in patients with chronic pulmonary sarcoidosis (NCT04064242)

This is a multi-national randomized double-blind placebo-controlled phase 2 study evaluating the safety, efficacy and tolerability of CMK389 in patients with chronic pulmonary sarcoidosis (212). Patients will receive a single intravenous (IV) infusion of CMK389 (vs. placebo) every 4 weeks for 16-weeks. Eligible subjects must be symptomatic, and on concomitant therapy with prednisone and methotrexate (or azathioprine) (212). Patients are excluded if they have significant pulmonary hypertension or extensive pulmonary fibrosis (> 20%) as determined by the grading system by Walsh et al. (213). The primary study outcome measure is change in FVC (212). Secondary outcome measures include exercise capacity [6MWD (214)], a composite index of pulmonary physiology (relative reduction in FVC > 10% or FEV1 = 10% or DLCo = 15%) and exercise capacity (relative reduction of 6MWD = 50 m), steroid sparing, and change in maximum and mean standardized uptake value (SUVmax and SUVmean) on PET/CT scan (116, 215). The projected study completion date is July 2023 with an expected enrollment number of sixty-six patients (212).

CMK389 is a fully human IgG1 monoclonal antibody directed against IL-18. Pre-clinical studies suggest that it selectively binds to and inhibits IL-18 activity. Significantly elevated levels of IL-18 have been found in serum and BAL fluids (BALF) of patients with pulmonary sarcoidosis and have been shown to play a significant role in the immunopathogenesis of the sarcoid granuloma formation (Figure 3) (216–219). IL-18 is a monocyte/macrophage derived pro-inflammatory cytokine which works synergistically with IL-12 to enhance IFN-γ production from Th1 cells (220, 221). On its own, IL-18 is weak at stimulating IFN-γ production, however, in conjunction with IL-12 it leads to enhanced IFN-γ production (221, 222). Studies in patients with pulmonary sarcoidosis showed that IL-18 also stimulates increased levels of IL-18 receptor (IL-18R) expression which activates AP1 and the transcription factor NF-κB leading to enhanced IL-2 gene expression and concomitant T-cell activation with an enhanced expression of Th1 cytokines (218, 219). IL-18 is produced as a procytokine which is cleaved intracellularly by caspase-1 to a mature biologically active form (219). Studies in Japanese patients with sarcoidosis suggest that IL-18 gene polymorphisms may be associated with an increased genetic risk of developing sarcoidosis (223).

#### RESOLVE-lung: A study to assess the efficacy and safety of Namilumab in patients with chronic pulmonary sarcoidosis (NCT05314517)

This is a randomized double-blind placebo-controlled phase 2 trial with open label extension (OLE) evaluating the safety and efficacy of anti-GM-CSF antibody (Namilumab) in patients with chronic pulmonary sarcoidosis (224). Participants will be randomized to receive a subcutaneous (SQ) injection of Namilumab (or placebo) every 4-weeks for a total of 26-weeks followed by an optional 28-week OLE of active study drug for patients who complete the 26-week double blind treatment period (224). As with CMK389, this trial targets patients with symptomatic chronic pulmonary sarcoidosis refractory to steroids. Patients with significant pulmonary fibrosis (=20%) or SAPH are excluded. The primary study outcome is a change in pulmonary function (assessed by the FVC). Other outcome measures include safety and tolerability of Namilumab, corticosteroid sparing effect, improvement in exercise tolerance (6MWD), overall improvement in HRQoL, and improvement in extrapulmonary organ manifestations of disease (ePOST score) (63). Similar to CMK389, improvement in lung parenchymal disease burden and extent of lung inflammation determined by changes in High-Resolution Computed Tomography Scans (HRCT) and SUVmean changes on PET scan will also be assessed (224). This study aims to enroll 100 participants worldwide and will run through January 2025.

Namilumab is a fully human IgG1 monoclonal antibody that binds with high affinity to GM-CSF and neutralizes its function (225, 226). It has been evaluated in patients with rheumatoid arthritis (227, 228), plaque psoriasis (229) and in hospitalized patients with severe COVID-19 pneumonia (230). While it was found to be effective in controlling symptoms and improving inflammation in patients with rheumatoid arthritis (227, 228) and severe COVID-19 pneumonia (230), it did not have any effect on patients with psoriasis (229). It is currently not FDA approval for any of these indications. GM-CSF is a hematopoietic growth factor produced by T-cells, alveolar macrophages, and fibroblasts (194). It has several proinflammatory effects and increased levels have been found in BALF of patients with active pulmonary sarcoidosis where it has been shown to correlate with disease activity (194, 195). The exact role of GM-CSF in the immunopathogenesis of the sarcoid granuloma is unclear, however, it has been shown to be involved in the alveolar cytokine network that promotes the formation and maintenance of granulomatous inflammation in patients with chronic sarcoidosis (Figure 3) (231).

Autoantibodies to GM-CSF (GM-CSFab) have been identified in patients with autoimmune pulmonary alveolar proteinosis (PAP) where they are known to be highly pathogenic (232–234). PAP is a rare life-threatening autoimmune disease characterized by accumulation of excess surfactant in the alveoli causing respiratory failure and predisposing to severe infections (232–234). There are several case reports of detection of GM-CSFab in sarcoidosis patients who subsequently developed PAP (235, 236) and there is concern that neutralizing GM-CSF activity in sarcoidosis patients may precipitate or unmask a co-existent PAP. Studies of Namilumab in patients with Rheumatoid arthritis did not reveal any evidence of lung damage, new-onset PAP, or evidence of opportunistic infections consistent with neutrophil dysfunction known to occur in patients with PAP, however, ongoing monitoring is warranted (228).

#### Efficacy and safety of intravenous Efzofitimod (ATYR1923) in patients with pulmonary sarcoidosis (NCT05415137)

This is the most advanced ongoing drug trial in pulmonary sarcoidosis. It is a multicenter randomized double-blind placebo-controlled phase 3 study evaluating the safety and efficacy of two IV doses of Efzofitimod (3 and 5 mg/kg) given every 4 weeks to patients with pulmonary sarcoidosis receiving stable doses of oral corticosteroids taken with or without additional immunosuppressant therapy (237). As with the other two trials above, a forced steroid taper is planned. The study plans to enroll 264 patients worldwide for completion in January 2025. Patients to be enrolled must be symptomatic from their disease (mMRC at least 1) and have evidence of disease associated impaired HRQoL (assessed by KSQ-Lung score < 70) (238). As with the other trials above, patients with advanced pulmonary fibrosis, clinically significant SAPH or other advanced and severe forms of extra-pulmonary sarcoidosis are excluded (237). The primary study outcome is steroid-sparing, and the secondary outcome measures are change in FVC and HRQoL as assessed by the Kings Sarcoidosis Questionnaire (KSQ)-Lung Score (239). This study is unique in incorporating HRQoL as a criterion for study enrollment and as a high-priority secondary outcome.

The exact mechanism of action of Efzofitimod in sarcoidosis is unclear. It is a novel immunomodulator that selectively binds the immunoregulatory receptor Neuropilin-2 (NRP2). Neuropilins (NRP) are multifunctional, single pass transmembrane, non-tyrosine kinase surface receptors expressed on all vertebrates (240, 241). Two isoforms (NRP1 and NRP2) have been identified and have been shown to be expressed in various subsets of innate and adaptive immune cells (macrophages, dendritic cells, T cells, B cells, and mast cells) where they regulate cell development, migration, recruitment, and modulate the overall immune response (240, 241). NRP2 is expressed on CD4 + effector T-cells, Treg cells, and in alveolar, bronchial, and intravascular macrophages (240). Immormino et al. found that NRP2 levels in alveolar macrophages were upregulated in a neutrophilic asthma model following challenge with an inhaled antigen/irritant suggesting that NRP2 regulates airway inflammation and plays a role in that disease (242). Two exploratory/preliminary studies in sarcoidosis showed that NRP2 is expressed in sarcoid granulomas (243, 244), however, its exact role in granuloma formation is unknown (241).

Efzofitimod is a fusion protein comprised of an immunomodulatory histidyl-tRNA synthetase (HisRS) domain fused to a human IgG1 Fc fragment. HisRS is an important pathogenic antigen (Jo-1 antigen) in autoimmune myositis (245–247) and patients with a history of Jo-1 antibody positivity or who screen positive for Jo-1 antibody are excluded from the study (237). The study will also monitor for the development of anti-Jo1 antibodies in study participants. A recently completed phase 2 study of Efzofitimod (ATYR1923) in patients with pulmonary sarcoidosis showed that this drug was safe and well tolerated and there was no signal of increased immunogenicity or formation of anti-Jo1 antibodies (study results published in abstract form) (132).

### Studies evaluating alternative treatment approaches in pulmonary sarcoidosis

#### PREDMETH: Effectiveness of methotrexate vs. prednisolone as first-line therapy for pulmonary sarcoidosis (NCT04314193)

This is a unique first of its kind randomized prospective trial designed to compare the efficacy and side-effects of monotherapy with methotrexate vs. prednisone as first-line therapy for patients with pulmonary sarcoidosis (248). Enrolled patients (treatment naïve pulmonary sarcoidosis patients) will be randomized to receive oral methotrexate (15 mg weekly to be increased to 25 mg weekly) vs. oral prednisone (starting at 40 mg daily to be tapered to 10 mg daily) for 24 weeks followed in both groups by another 18-month period of regular care (25, 248). Patients will be required to perform hospital visits and in addition, will perform weekly home spirometry and record symptoms and medication side-effects *via* a home monitoring application. The primary study objective is to investigate the efficacy and tolerability of methotrexate as first-line therapy in patients with pulmonary sarcoidosis compared to prednisone (25). Secondary objectives are to gain more insights in response to therapy in individual patients by home spirometry and PROMs including the Fatigue Assessment Scale (FAS), the King’s Sarcoidosis Questionnaire (KSQ), Global Rating of Change Scale (GRoC), Chronic Respiratory Questionnaire (CRQ), Patient Experience and Satisfaction with Medication Questionnaire (PESaM) and Euroqol-5D-5L questionnaire (EQ-5D-5L) (25). Several biomarkers including (sACE, sIL-2R, Monocyte and T-lymphocyte numbers) will also be examined to find predictors of response to therapy, disease progression and chronicity, and to further improve understanding of the underlying disease mechanism.

The primary study endpoint is change in hospital measured FVC between baseline and 24 weeks (25). The secondary study endpoints include time to pulmonary improvement measured by home-spirometry (home-FVC), percentage of patients with = 5 and = 10% improvement or decline in FVC and DLCO at 4, 16, and 24 weeks, improvement or decline in HRQoL, experiences and satisfaction with medications, severity and impact of side-effects compared between prednisone and methotrexate, adherence to treatment schedule, and number of patients who discontinue or switch medication (25). Changes in biomarkers over time, and correlation between biomarkers and clinical parameters will also be evaluated as exploratory endpoints (25).

This study is ongoing in The Netherlands and results are expected in January 2025. If this study confirms the hypothesis that methotrexate is as effective as prednisone as first-line therapy for pulmonary sarcoidosis, but with fewer side effects, it will provide an important “steroid alternative” regimen for a small but not insignificant population of patients for whom a “steroid-alternative” or “steroid-avoidance” regimen is essential.

#### QUIDOSE: A randomized controlled trial of hydroxychloroquine combined with low-dose corticosteroid in pulmonary sarcoidosis (NCT05247554)

This is another large phase 3 RCT that is designed to evaluate the hypothesis that a regimen with lower cumulative prednisone doses will be as effective as one with higher cumulative prednisone doses with less toxicity and better HRQoL measures (34, 35). Patients will be randomized to receive Hydroxychloroquine 400 mg daily for 6-months combined with low-dose prednisone 20 mg daily for 1 month followed by 10 mg daily for 5 months (cumulative prednisone dose of 1,820 mg) vs. initial monotherapy therapy with prednisone 40 mg daily for 4 weeks followed by 30 mg daily for 2 weeks, then 20 mg daily for 2 weeks, then 15 mg daily for 2 weeks then 10 mg daily for 14 weeks for a total 6-month cumulative dose of 2,870 mg of prednisone (249). The primary study outcome is difference in percent predicted FVC between inclusion and 6-months in the two groups.

Hydroxychloroquine is an antimalaria drug that has been shown in case reports and small studies to be effective in patients with cutaneous and pulmonary sarcoidosis, sarcoidosis associated hypercalcemia, and in select patients with neurosarcoidosis (250–254). The mechanism of action of hydroxychloroquine in sarcoidosis is varied. It has anti-inflammatory properties and has been shown to interfere with antigen presentation, prevent T-cell activation, inhibit Toll like receptor signaling, and reduce production of inflammatory cytokines by T and B cells (255, 256). It is very well tolerated with only minimal gastrointestinal side effects reported (257).

Hydroxychloroquine has been associated with retinal toxicity and patients with known retinopathy or maculopathy are excluded from this study (258, 259). This study is based in France and the planned completion date is March 2024.

#### SARCORT: Efficacy and Safety of Two Glucocorticoid Regimens in the Treatment of Sarcoidosis (NCT03265405)

is an ongoing randomized parallel assignment open label study in India evaluating the safety and efficacy of a treatment regimen of low dose prednisone (given as 20 mg/day for 8 weeks, followed by 15 mg/day for 8 weeks, 10 mg/day for 4 weeks and 5 mg/day for 4 weeks) vs. medium dose prednisone (given as 40 mg/day for 4 weeks, followed by 30 mg/day for 4 weeks, 20 mg/day for 4 weeks, 15 mg/day for 4 weeks, 10 mg/day for 4 weeks and 5 mg/day for 4 weeks). Participants in both groups will be followed for evidence of disease relapse at the end of 18-months (260). The study authors hypothesize that a higher initial dose of prednisone will be more effective in preventing post treatment relapse than a lower prednisone dose. Enrolled patients will have symptomatic pulmonary sarcoidosis with evidence of impaired pulmonary function (FEV1 < 80%) and/or active extra-pulmonary sarcoidosis requiring treatment. The primary study outcome is the proportion of subjects with a relapse or treatment failure at the end of 18-months. Secondary outcome measures include difference in mean time to relapse, proportion of patients with disease stabilization, improvement, or resolution of disease at 18-months, and the difference in mean FVC at the end of the 6-months. Other secondary outcomes of interest include cumulative prednisone dose, steroid toxicity/adverse effects and HRQoL measured using the Sarcoidosis Health Questionnaire (SHQ) (261) and the FAS.

It is to be noted that patients enrolled in both the SARCORT (260) as well as QUIDOSE (249) trials could potentially be exposed to cumulative steroid doses shown in prior trials to be associated with reduced HRQoL and potentially increased toxicity (34, 35, 262). Patients randomized to the low-dose prednisone arm in SARCORT will receive a 6-month cumulative prednisone dose of 2,380 mg vs. 3,360 mg for patients randomized to the medium dose prednisone arm (260). Participants in the QUIDOSE trial will likewise be exposed to cumulative prednisone doses of 1,820 mg (low dose arm) vs. 2,870 mg in the steroid mono-therapy arm (249). While there is no definition for what quantity of cumulative prednisone doses qualify as “high,” “medium,” or “low” dose, Judson et al. (35) found that patients who received > 500 mg of prednisone in the preceding year (“high dose prednisone group”) had worse HRQoL (35). Cox et al. also reported similarly reduced HRQoL in patients prescribed prednisone, however, the cumulative prednisone doses were not reported (262). More recently, Broos et al. (34) observed that there was no significant difference in the number of patients who experienced an exacerbation or relapse during tapering in patients who receive a 12-month cumulative prednisone dose of 4,000 mg or more (“high dose prednisone group”) vs. those who received a lower 12-month cumulative dose (“low dose prednisone group”) (34). Additionally, patients who received higher prednisone doses had more toxicity and increased weight gain, and there was no correlation between prednisone dose and pulmonary function as assessed by FVC (34). These studies serve to emphasize the need to report cumulative prednisone doses in all sarcoidosis trials; and importantly also, to clearly define what constitutes a low vs. high dose steroid regimen in cumulative prednisone doses and not just as a final prednisone dose of < 10 mg/day.

### Recently completed drug trials in pulmonary sarcoidosis

There have been several recently completed early phase trials of novel therapeutic agents in sarcoidosis.

#### Nicotine treatment for pulmonary sarcoidosis: A clinical trial pilot study (NCT02265874)

This randomized double-blind placebo-controlled phase 1b/2a pilot study evaluated the safety, efficacy, and tolerability of transdermal nicotine (vs. placebo) given as a daily 21 mg patch for 24 weeks in patients with active symptomatic pulmonary sarcoidosis receiving a maximum daily dose of 10 mg prednisone (or prednisone equivalent) without any concomitant 2nd or 3rd line therapy (211, 263).

Several epidemiologic studies show that cigarette smokers and smokeless tobacco users have a twofold reduced risk of developing sarcoidosis (264–267). Nicotine has a potent immunomodulatory activity on T-cell-mediated inflammation *via* α7 nicotinic cholinergic receptors (α7nAChR) which signal through the JAK-STAT pathway (268–272). At high concentrations, nicotine suppresses antigen-mediated TNF-α production (273), increases the suppressive action of Treg cells (274), and suppress Th1- and Th17-type immune responses (269, 270). These responses appear to be more pronounced in the lungs and are thought to create a microenvironment that results in inhibition of granuloma formation (211, 263, 275).

This study was conducted between October 2015 and January 2019 in two centers in the U.S. and enrolled 49 patients with pulmonary sarcoidosis randomized to receive transdermal nicotine vs. placebo for 24 weeks (211). Study participants were never smokers or former smokers and were required to be non-smoking for at least 6-months prior to study enrollment (211). Patients were followed with serial PFTs, quantitative lung texture score (based on computed tomography (CT) texture analysis), Fatigue Assessment Score (FAS), and HRQoL measures (263). Overall, Nicotine treatment was well tolerated and safe. There was a clinically significant (2%, 70 cc) improvement in FVC and a trend to improvement in FAS. There was no change in HRQoL scores or change in radiographic burden of disease as assessed by serial CT texture analysis (211). Similar to findings from an earlier study (276), Nicotine was found to be non-addictive (211).

While the results of this study appear positive, it is important to note that it was a small study and larger Phase 3 trials to further evaluate the role of transdermal nicotine as a therapeutic option for sarcoidosis are needed. To ensure a secure place for Nicotine as a treatment option in sarcoidosis, future studies will need to demonstrate improvements in HRQoL and in pulmonary parenchymal disease burden in addition to improvements in pulmonary function. It will also be important to continue to document that prolonged use of Nicotine remains non-addictive. The study was performed in relatively mild patients, with only a quarter on low dose prednisone at time of study entry. Several study participants were newly diagnosed treatment naïve patients (211). A place for nicotine as a first-, second- or third line treatment option in sarcoidosis will need to be determined.

#### Phase II investigation of antimycobacterial (CLEAR) therapy on progressive, pulmonary sarcoidosis (NCT02024555)

This recently completed phase 2b multi-center randomized double blind placebo controlled trial evaluated the role of antimicrobial therapy given as a concomitant regimen of Levofloxacin 500 mg daily, Ethambutol 1,200 mg daily, Azithromycin 250 mg daily and Rifampin 600 mg daily (or Rifabutin 300 mg daily) for 16 weeks (CLEAR regimen)—to patients with chronic progressive pulmonary sarcoidosis (CPPS) (106). Participants were required to have evidence of parenchymal or nodal disease on chest radiograph (CXR) and met criteria for CPPS if they had any of the following: (1) 5% decline in absolute percentage predicted of FVC or DLCO on serial measurements over 24-months; (2) radiographic disease progression on CXR observed on a side-by-side comparison; or (3) decline in dyspnea score, as measured using the transition dyspnea index (TDI) (106, 124). Patients with end stage fibrotic pulmonary disease [Scadding stage IV on CXR (277)] were excluded. Patients were allowed to continue their baseline immunosuppressive regimen, however, patients on prednisone (or equivalent) doses > 40 mg/day or receiving biologic medications within 6-months of the study were excluded (106).

The study enrolled 97 patients (52% female and 29% African American) across four sites in the U.S. 49 patients were randomized to receive CLEAR regimen and 48 patients to Placebo. Each patient received 8 weeks of four drugs (or matching placebo) followed by 8 weeks of two drugs (or matching placebo) based on individual tolerance and toxicity profile during the first 8 weeks (106). The primary endpoint was change in FVC, however, change in 6MWD, HRQoL, adverse events grades and ESAT-6-specific immune responses were also reported. Overall, this study did not find any benefit of CLEAR therapy over placebo in patients with CPPS (106). Patients on CLEAR therapy had a significant decline in ESAT-6 immune responses, however, there was no corresponding change in FVC or 6MWD at the end of 16-weeks (106). Furthermore, patients randomized to active intervention reported worse HRQoL scores than patients on placebo (106).

The results of this study are in complete contradiction with results of prior studies that provided evidence suggestive of a potential benefit of CLEAR therapy in patients with pulmonary (278) and cutaneous (279) sarcoidosis. The reasons for this discordance are unclear and several plausible theories have been advanced (106). While it is entirely possible that the negative trial results may be accounted for by the presumption that mycobacteria has no role in the etiopathogenesis of sarcoidosis (and thus use of antimycobacterial agents will have no effect), the authors acknowledge that methodological flaws in the study design provide more likely explanations (106). These design flaws will hopefully serve to guide future trials (19). One of the major concerns surrounding the negative study results center on patient selection. It is speculated that by selecting patients with evidence of disease progression over 24 months (in lieu of a shorter duration), the authors may have inadvertently selected for patients with chronic stable disease for whom additional therapy provided no added benefit while incurring additional burden (and negative HRQoL impact) of taking additional pills with increased toxicities. In further support of this point is the observation that patients were selected based on CXR findings without other biomarker evidence of disease activity. Several studies have shown that CXRs are insensitive at detecting active disease in patients with sarcoidosis, and high-resolution CT (HRCT) and 18F-FDG PET scans have been advocated for this purpose (62, 118–122). The authors note that use of 18F-FDG PET scans to detect active disease was not common practice at the time of study design (106). Another concern raised is the absence of a forced steroid or other immunosuppressant (IST) taper (106). Study participants were continued on stable doses of steroids and IST, and this may have blunted the ability to determine the additive contribution of CLEAR therapy while negatively impacting HRQoL due to an overall increased pill burden.

Regardless of the reason for the results obtained, it is important to note that they do not reflect on all antibiotic trials in sarcoidosis. A recently concluded non-controlled open label phase 1b study of Azithromycin 250 mg taken once daily for 3-months in 21 patients with pulmonary sarcoidosis presenting with chronic cough found that Azithromycin led to improved cough metrics and HRQoL measures (280). Patients in this latter study were maintained on corticosteroid monotherapy. Larger phase 2b RCTs of Azithromycin are planned. Perhaps, trials with a reduced pill burden and forced corticosteroid or concomitant IST taper may yield different results.

##### Other recently completed studies in pulmonary sarcoidosis

Several other studies have been recently completed in patients with pulmonary sarcoidosis. These studies evaluated the role of Canakinumab (a fully human anti-IL1β monoclonal antibody) (NCT02888080) (281); and RCI (NCT03320070) (282) in patients with pulmonary sarcoidosis. Canakinumab is a novel therapeutic agent and the role of IL-1 in sarcoid granuloma formation has been addressed above.

RCI is a complex mixture of prolonged-release adrenocorticotropic hormone (ACTH) and other pituitary peptides (283). It has a complex multi-mechanistic action in sarcoidosis that is distinct from that of corticosteroids (283) and several studies have found RCI to be corticosteroid sparing in sarcoidosis and other autoimmune diseases (126, 133, 283, 284). One of the key outcomes of the RCI phase 4 study (PulSAR trial) is the effect of RCI on the Sarcoidosis Treatment Score (STS) which is a composite measure that captures multiple facets of pulmonary sarcoidosis including pulmonary function (FVC and DLCO), fatigue, HRQoL, fatigue, corticosteroid taper and lung parenchymal changes on HRCT scan (285). Although RCI has a mechanism of action that is distinct from corticosteroids, its side effect profile is identical to that of corticosteroids, and it is important that patients on RCI are rapidly tapered off corticosteroids to minimize toxicity (286).

Friedman et al. also recently published results from a recently concluded small phase 1 proof of concept study evaluating the role of Tofacitinib given as a 5 mg oral pill twice daily for 16-weeks in 5 patients with pulmonary sarcoidosis (128). They found that addition of Tofacitinib allowed 3 out of 5 (3/5) patients with steroid refractory pulmonary sarcoidosis to taper their prednisone to <5 mg daily (128). Additionally, patients reported improved symptom burden and HRQoL scores (128). One patient was withdrawn from the study due to worsening neurosarcoidosis despite stable pulmonary disease. This is a very small study and larger studies are needed.

## Studies with a focus on extra-pulmonary and multi-organ sarcoidosis

### Sarilumab in patients with glucocorticoid-dependent sarcoidosis (NCT04008069)

This is a small single center phase II study that is planned to enroll 15 patients with steroid refractory pulmonary and extra-pulmonary sarcoidosis randomized to Sarilumab vs. placebo (287). Sarilumab is a recombinant humanized IgG1monoclonal antibody directed against IL-6 receptor (288–290). It is FDA approved for the treatment of moderate to severe rheumatoid arthritis refractory to TNFi therapy and other disease modifying anti-rheumatic drugs (DMARDs) (288–290). The well-established role of IL-6 in the formation of the sarcoid granuloma (Figure 3) (157, 291, 292) and evidence that levels of IL-6 correlate with sarcoidosis disease activity and severity (293–295) make it a unique target for sarcoidosis.

All enrolled patients will receive Sarilumab given as a subcutaneous injection every 2 weeks for 16 weeks and will undergo a forced steroid taper. Patients who successfully taper off steroids by week 16, will be randomized to receive continued SQ injections of Sarilumab vs. placebo for an additional 12-weeks. The primary outcome of interest is flare-free survival of Sarilumab treated patients defined as ability to remain off prednisone and other therapies while on Sarilumab. This study targets patients with steroid refractory (prednisone dose 10–60 mg/day) and multi-organ disease. It has a unique study design that incorporates patients with pulmonary sarcoidosis enriched for presence of non-life-threatening multi-organ disease. In addition to patients with pulmonary sarcoidosis, the target population includes patients with active glucocorticoid-dependent sarcoidosis affecting the lymph nodes, liver, kidneys, spleen, bone, soft tissues, skin, and/or eyes while excluding patients with fibrotic pulmonary sarcoidosis (FPS), CNS sarcoidosis, and CS (287). Furthermore, the primary study outcome of flare-free survival could also be considered a correlate of TTCW, and the investigators have incorporated drop-out from placebo arm as a component of this primary outcome measure (287). As expected, the secondary outcome measures assess effect of therapy on multiple organs and include change in pulmonary function (FVC, FEV1), extrapulmonary physician organ severity tool (ePOST) score (63), SASI and size of skin lesions (for cutaneous sarcoidosis), 68/66 Joint evaluation (296), and kidney and renal function. Change in fatigue scores using the Functional Assessment of Chronic Illness Therapy-Fatigue subscale (FACIT-F) (297, 298) are also monitored, however, no other specific HRQoL PROMs are mentioned. This study is projected to be completed in July 2025 and will address a much-needed niche in sarcoidosis (287).

### Phase I hypothesis generating study evaluating the role of Tofacitinib in cutaneous sarcoidosis (NCT03793439)

Tofacitinib is a JAK1/JAK3 inhibitor that is currently approved for the treatment of Rheumatoid arthritis refractory to conventional DMARD therapy (299, 300). Anecdotal case reports of successful treatment of patients with refractory cutaneous sarcoidosis using JAK inhibitors (301–303), and immunological evidence of JAK-STAT pathway activation in patients with sarcoidosis (186, 304) has raised interest in the possible use of JAK inhibitors in patients with sarcoidosis (18).

Damsky et al. (305) recently published an open-label study of ten patients with cutaneous sarcoidosis. The authors found that treatment led to significant improvement of skin lesions using the previously validated cutaneous sarcoidosis activity and morphology instrument (CSAMI) scoring system. In detailed studies, the skin changes were associated with significant changes in the inflammatory profiles. There was some evidence of response for extra-cutaneous manifestations. However, the number of patients with extra-cutaneous disease were small and the end points were poorly described. Nevertheless, this was an important validation of the original case reports (305).

### EFIRTES: Efficacy of remission-induction regimen with infliximab for severe extrathoracic sarcoidosis (NCT03704610)

This study conducted in France is focused on patients with extra-pulmonary multi-organ sarcoidosis (306). It is a randomized placebo-controlled phase 3 multi-center study designed to assess the efficacy of a loading dose of infliximab given as a 5 mg/Kg infusion (vs. Placebo) every 2-weeks for two doses (day 0 and 15) in patients with active or recurrent multi-organ sarcoidosis despite ongoing treatment with a first-line immunosuppressive drug. Patients are randomized to receive a loading dose of infliximab (vs. placebo) in the first 2 weeks, followed in both arms by open label injection of infliximab for a total of 3 additional doses in the intervention arm and 5-additoinal doses in the placebo arm. The primary outcome measure is the percentage of patients who have an ePOST score < 1 in all organs (including absence of hypercalcemia) at week 6 regardless of the corticosteroid dosage received, and the secondary outcome measures include the percentage of patients who completed a forced steroid taper while on active intervention and who have an ePOST score < 1 without any evidence of relapse or treatment failure (306). As with the two other studies referenced above, there is no specific mention of HRQoL assessments.

It is to be noted that whilst there is broad consensus that HRQoL is an important and relevant outcome in sarcoidosis research (5, 22, 113), in the current landscape of pharmaceutical trials, HRQoL does not appear to have been accorded a place as a top-tier endpoint and is often only given cursory attention as a secondary endpoint. Additionally, many studies refer to the measurement of HRQoL but do not define if they are measuring all dimensions of HRQoL using condition specific measures or generic measures or in fact health status and symptom burden rather than HRQoL *per se* (26). This lack of attention and transparency around the choice of PROMs will need to be addressed in future studies. Having a dedicated set of core outcome measures that are both organ- and systemic-disease specific will also help to address this.

## Studies in severe forms of disease: Fibrotic pulmonary sarcoidosis/cardiac sarcoidosis

### Studies in fibrotic pulmonary sarcoidosis

About 10–30% of sarcoidosis patients develop progressive fibrotic disease and it is associated with a significantly increased morbidity and mortality (307, 308). The immuno-etiologic factors driving the transition from chronic sarcoidosis to fibrotic disease are poorly understood (200). While most experts believe that ongoing unbridled granulomatous inflammation is the key driver of fibrosis (200, 309, 310), recently published work showed that fibrotic foci (FF) known to be the hallmark drivers of fibrosis in idiopathic pulmonary fibrosis (IFP) were also present in patients with FPS, and that the gene and protein expressions were similar despite differing initiation pathways (311). This finding suggests that management of patients with FPS will need to address loss of lung function due to both the ongoing inflammation as well as to progressive fibrosis. Thus, studies pointing toward the use of a combined anti-fibrotic and immunosuppressive agent such as mycophenolate mofetil (312, 313) which has demonstrated global significant improvement in lung function in patients with systemic sclerosis, may be worth investigating (314, 315). This is in contradistinction to (or perhaps in addition to) anti-fibrotic agents which demonstrated only deceleration of progressive fibrosis without improvement in other important disease parameters such as HRQoL, cough, dyspnea, or systemic manifestations (316). Patients with FPS express various clinical phenotypes typified in some patients by alternating periods of rapid disease progression followed by periods of stability and in others by a slowly progressive indolent disease course (317, 318).

The INBUILD trial (319) revealed that Nintedanib (an oral intracellular tyrosine kinase inhibitor) was associated with a statistically significant deceleration in disease progression and loss of lung function in patients with progressive fibrosing interstitial lung disease (PF-ILD) regardless of the radiographic pattern of disease or the underlying etiology (319). Since that study was published, the term PF-ILD has now been replaced with progressive pulmonary fibrosis (PPF) (320) and a recently published systematic review and GRADE based meta-analysis of available data regarding the use of Nintedanib in patients with PPF found that a generalized conclusion about the effects of Nintedanib in all the various subtypes of PPF could not be made (321). A *post hoc* analysis of the INBUILD study found that of the 663 patients enrolled, only 12 patients (<2% of the study population) had sarcoidosis with evidence of PPF (322). 4 of these patients received Nintedanib and 8 received placebo (321, 322). Further evaluation of these 12 patients showed that there was no consistent effect of Nintedanib on lung function (321). Furthermore, none of these patients contributed data to all-cause mortality, adverse treatment effects, or to time to first exacerbation or death (321). When grouped into radiographic patterns, only sarcoidosis patients with a radiographic UIP pattern [as described by Raghu et al. (320)] derived benefit from Nintedanib; however, there were only three such patients and two of them were randomized to placebo (321). Consequently, the role of Nintedanib or other anti-fibrotic therapy in FPS remains unknown.

There is currently no ongoing trial evaluating the role of anti-fibrotic therapy in patients with FPS. A small recently completed phase 2 study that evaluated the role of pirfenidone (vs. placebo) in patients with FPS enrolled 16 patients and was underpowered to determine pirfenidone efficacy (131). In that study, only patients with > 20% fibrosis on HRCT or DLCO < 40% met the clinical end point of time to clinical worsening (TTCW), which was defined as death, lung transplant or > 10% absolute drop in percent predicted FVC at the end of the study period (131).

Patients with FPS present varied radiographic profiles which may correlate with lung function (317, 318, 323). In evaluating the role of anti-fibrotic therapy in sarcoidosis, it will be important to determine how the radiographic pattern of disease [UIP vs. non-UIP pattern (320)] interacts with DLCO in determining which patients respond to therapy. It will also be important to clearly define what disease progression means in patients with FPS as not all the FPS patients with clinical or radiographic worsening will warrant anti-fibrotic therapy (320). Finally, the role of anti-fibrotic therapy in preventing or prolonging time to “acute exacerbation” of disease in FPS will need to be determined. Baughman and Lower (324) showed that patients with FPS experience a high frequency of acute worsening events or “acute exacerbations” (about three per year), however, this was more common in patients who had evidence of bronchiectasis on HRCT (324). A recently reported study of Roflumilast in patients with FPS, notes that these exacerbations were characterized by increased cough and sputum production; and use of the phosphodiesterase 4 (PDE-4) inhibitor (Roflumilast) was associated with a lower rate of acute events than placebo (129). A prior report also found that “acute exacerbations” in patients with FPS responded to short courses of prednisone therapy (325). While Nintedanib increased the time to first exacerbation in patients with both IPF and PPF (326, 327), the mechanism of exacerbation in patients with IPF and other forms of PPF has not been associated with underlying bronchiectasis and particularly in IPF, has not shown consistent response to corticosteroids (328–330).

### Studies in cardiac sarcoidosis

There are several ongoing interventional drug trials evaluating new molecules and therapeutic treatment approaches in patients with CS.

RESOLVE-Heart (NCT05351554) is a randomized double-blind placebo-controlled phase 2a study that is planned to evaluate the safety, efficacy, and tolerability of Namilumab in patients with active CS (331). It is a hybrid study with two planned cohorts. Patients enrolled in Cohort A will be randomized to receive SQ Namilumab vs. matching placebo, while all patients in cohort B (open Cohort) will receive active intervention. All study participants will continue their stable doses of prednisone and other background IST for the study duration (30 weeks). The primary study outcome is the safety and tolerability of Namilumab (measured by the incidence and severity of treatment emergent adverse events, serious adverse events, and adverse events leading to discontinuation), however, the effect of intervention on cumulative arrythmia burden, HRQoL, ability to achieve steroid taper, changes in left ventricular ejection fraction (LVEF) and other echocardiographic variables will also be assessed. Patients will have a cardiac PET scan prior to and after enrollment and mean changes in SUVmax will be assessed. The study is expected to begin enrollment soon and expected completion date is January 2024. The mechanism of action and rationale for use of Namilumab in sarcoidosis has been discussed above.

MAGiC-ART (NCT04017936) is an open label Phase 2 pilot study evaluating the role of IL-1 blockade in patients with CS (332, 333). Study participants will be randomized to receive daily subcutaneous injections of Anakinra plus standard of care (vs. standard of care only) for 4 weeks (332, 333). The primary study outcome is change in plasma C-reactive protein (CRP) at 28-days, and secondary outcome measures include change in cardiac inflammation and fibrosis measured by cardiac PET scan and cardiac MRI, and number of serious cardiac events measured by the sum of hospitalizations and deaths from cardiac causes from baseline to 28-days (333). Patients to be enrolled must have evidence of abnormal myocardial uptake and an elevated CRP at baseline (332). In addition to its role in sarcoid granuloma formation (Figure 3), IL-1 has been shown to play a significant role in the pathophysiology of heart disease including ischemic heart disease and heart failure (334, 335). Patients with clinically active CS, and evidence of active inflammation on FDG-PET scans have demonstrated evidence of active inflammasomes with IL-1β activity in biopsies of granulomatous lesions obtained prior to cardiac surgery (336). Anakinra is a recombinant human IL-1 receptor antagonist that is currently FDA approved for the treatment of Rheumatoid arthritis (337). Several studies suggest that treatment with Anakinra has been associated with a reduced incidence of heart failure in patients with myocardial infarction, and an improved cardiorespiratory fitness and HRQoL in patients with systolic heart failure (338–340). There are no prior trials of Anakinra in sarcoidosis. This study is projected to enroll 28 participants and is planned for completion in December 2023 (333).

CHASM CS-RCT (NCT03593759) is a large phase 3 non-inferiority multi-center multi-national RCT designed to evaluate the optimal initial treatment strategy for patients with active CS (24, 341). Newly diagnosed treatment naïve CS patients will be randomized to initiate treatment with prednisone monotherapy or combination therapy with prednisone and methotrexate given for the first 6-months after diagnosis (24). The primary study endpoint is the degree of myocardial fibrosis and scarring measured by the summed perfusion rest score (SPRS) obtained on the 6-month follow-up PET scan (24, 341). Presence and extent of fibrosis (SPRS) was chosen over suppression of inflammation (SUV mean/max) as a primary endpoint consistent with the primary aim of therapy in sarcoidosis being to prevent end organ damage due to fibrosis and not merely to suppress inflammation (24). Secondary study endpoints include mortality, cardiovascular hospitalizations, medication adverse events, glucocorticoid toxicity, generic and disease specific HRQoL, extra-cardiac disease activity, ventricular arrhythmia burden, left, and right ventricular ejection fractions and FDG uptake, several biomarkers, and the burden of late gadolinium enhancement on cardiac MRI (24, 341). This is the largest study in CS and will provide very valuable information on the optimal initial therapeutic option in CS. The rationale for this study is based on findings from several small studies suggesting that CS patients treated with an initial regimen of corticosteroids and IST at diagnosis had better outcomes (reduced rate of relapse and improved or stable LVEF) than those treated with steroid monotherapy (342, 343). Furthermore, these differences persisted even if other IST were subsequently added (342).

J-ACNES (UMIN 000025936) is the Japanese Antibacterial Drug management for CS, an ongoing multicenter open-label RCT designed to investigate the effect of antibiotic treatment in addition to standard corticosteroid therapy in patients with CS (344). The primary objective of this trial is to investigate the clinical benefit and safety of antibiotic therapy (clarithromycin plus doxycycline) in addition to corticosteroids in patients with CS and is based on findings from several studies identifying *Propionibacterium acnes* in sarcoid granulomas of myocardial tissue (344). Newly diagnosed treatment naïve CS patients with evidence of abnormal myocardial uptake will be randomized to receive either corticosteroid therapy plus antibiotic (ABD group) or corticosteroids alone (standard group) for 6-months followed by an observation period of 4.5 years during which all study participants will receive standard corticosteroid therapy (344). The primary study endpoint is change in total SUV at 6 months vs. baseline. Secondary study endpoints include frequency of corticosteroid dose escalation, change in the maximum and mean SUV and change in LVEF at 6- and 12-months, a composite of major adverse cardiovascular events (cardiovascular death, lethal arrythmias and hospitalization for heart failure) at 6, 12, 36, and 60 months, frequency of adverse events and treatment discontinuation, and change in various biomarkers including sACE, lysozyme level, and sIL-2R levels at 6 and 12-months (344). The rate of reduction in plasma *P. acnes* lipoteichoic acid concentration (ACNEX) at 6 and 12 months will also assessed (344). A minimum of 80 patients will be enrolled, however, the final sample size will be dependent on findings from a planned interim analysis (344).

## Looking ahead to improve future clinical drug trials

This is an exciting time for therapeutic trials and trials evaluating new treatment approaches in sarcoidosis. For the first time in over two decades, preliminary guidance for the diagnosis and treatment of sarcoidosis have been published and work has begun on defining a set of core outcome measures that can be uniformly applied in clinical trials (1, 5, 22). Additionally, for the first time since 2014 (105, 106), there is a renewed focus on evaluating new molecules and new therapeutic targets for sarcoidosis. While some much-anticipated trials and therapeutic regimens have returned negative results (106), it is important to understand that these trials teach as much or more about the disease process and sarcoidosis clinical trial conduct than those with positive results. Ongoing challenges to clinical trials abound, yet these are not insurmountable.

The outlook to improve future drug trials in sarcoidosis should look to:

– Enhance and diversify subject recruitment and retention by eliminating all real and perceived barriers to trial participation. This includes an active outreach to underserved and minority communities.
– Clearly define target cohorts and optimize mechanisms of ascertaining who has active disease prior to trial enrollment. Concomitant with this is the need to clearly define physiologic and clinical endpoints of disease that are consistent with the pathobiology of sarcoidosis and at the same time meaningful to patients.
– Determine a core set of organ-specific HRQoL and patient-centered outcome measures that will enable comparison of trial results across studies and minimize redundancy and waste of resources.
– Optimize clinical trial designs to enrich study populations of pulmonary sarcoidosis for other organs of interest without detracting from pulmonary specific endpoints.
– Build in protocols that address and manage steroid toxicities for studies whose primary outcome is “steroid-sparing”; while at the same time seeking new and safer endpoints that capture clinical deterioration and lack of response to therapy without incurring significant steroid toxicities.
– Prioritize studies evaluating the appropriate management of FPS. There is a high morbidity and mortality associated with FPS, yet this group of patients are routinely excluded from clinical trials. Recent findings of the similarities between fibrosis in sarcoidosis and IPF and increasing availability and efficacy of anti-fibrotic therapy should serve as a catalyst for further attention to this severe disease manifestation. The various phenotypes of FPS need to be elucidated and optimal treatment approach for these patients determined.
– Finally, while this manuscript was limited to only pharmacologic intervention, it is to be noted that there have been advancements in non-pharmacological management of sarcoidosis, and these will need to be studied alongside the pharmacologic interventions for a more holistic approach to patient care.

Having come this far, the sarcoidosis community needs to maintain an unflinching resolve to ensure that large scale well designed RCTs be performed for all future therapeutic interventions and that each subsequent/updated guideline reflect an improved grade of evidence for guideline recommendations.

## Conclusion

Over 50% of patients with sarcoidosis require treatment for their disease yet none of the medications currently used for sarcoidosis treatment has been rigorously studied in large RCTs (5, 17) and evidence supporting use of these medications is weak in the best of cases.

There are several ongoing RCTs evaluating new therapeutic molecules, novel therapeutic targets and steroid alternative treatment regimens in sarcoidosis with several yielding positive early phase results (211).

While these ongoing trials and future potential trials face significant challenges, it is important to note that these challenges are not insurmountable, and several potential solutions have been proffered in this manuscript.

## Author contributions

All authors wrote, edited, reviewed, and contributed key concepts to the manuscript. All authors approved the submitted version.
